# The structural basis for HIV-1 Vif antagonism of human APOBEC3G

**DOI:** 10.1038/s41586-023-05779-1

**Published:** 2023-02-08

**Authors:** Yen-Li Li, Caroline A. Langley, Caleigh M. Azumaya, Ignacia Echeverria, Nicholas M. Chesarino, Michael Emerman, Yifan Cheng, John D. Gross

**Affiliations:** 1 Department of Pharmaceutical Chemistry, University of California, San Francisco, CA USA; 2 Divisions of Human Biology and Basic Sciences, Fred Hutchinson Cancer Center, Seattle, WA USA; 3 Molecular and Cellular Biology Graduate Program, University of Washington, Seattle, WA USA; 4 Fred Hutchinson Cancer Center, Electron Microscopy Shared Resource, Seattle, WA USA; 5 Department of Cellular and Molecular Pharmacology, University of California, San Francisco, CA USA; 6 Quantitative Bioscience Institute, University of California, San Francisco, CA USA; 7 Department of Bioengineering and Therapeutic Sciences, University of California, San Francisco, CA USA; 8 Department of Biochemistry and Biophysics, University of California, San Francisco, CA USA; 9 Howard Hughes Medical Institute, University of California, San Francisco, CA USA

**Keywords:** Virus-host interactions, Retrovirus, Cryoelectron microscopy, Restriction factors

## Abstract

The APOBEC3 (A3) proteins are host antiviral cellular proteins that hypermutate the viral genome of diverse viral families. In retroviruses, this process requires A3 packaging into viral particles^1–4^. The lentiviruses encode a protein, Vif, that antagonizes A3 family members by targeting them for degradation. Diversification of A3 allows host escape from Vif whereas adaptations in Vif enable cross-species transmission of primate lentiviruses. How this ‘molecular arms race’ plays out at the structural level is unknown. Here, we report the cryogenic electron microscopy structure of human APOBEC3G (A3G) bound to HIV-1 Vif, and the hijacked cellular proteins that promote ubiquitin-mediated proteolysis. A small surface explains the molecular arms race, including a cross-species transmission event that led to the birth of HIV-1. Unexpectedly, we find that RNA is a molecular glue for the Vif–A3G interaction, enabling Vif to repress A3G by ubiquitin-dependent and -independent mechanisms. Our results suggest a model in which Vif antagonizes A3G by intercepting it in its most dangerous form for the virus—when bound to RNA and on the pathway to packaging—to prevent viral restriction. By engaging essential surfaces required for restriction, Vif exploits a vulnerability in A3G, suggesting a general mechanism by which RNA binding helps to position key residues necessary for viral antagonism of a host antiviral gene.

## Abstract (web summary)

The authors report the cryo-EM structure of human A3G bound to HIV-1 Vif, and the hijacked cellular proteins that promote ubiquitin-mediated proteolysis, suggesting how Vif antagonizes A3G by intercepting it to prevent viral restriction.

## Main

The APOBEC3 (A3) proteins are host cytosine deaminases with the capacity to mutate viral genomes across many different viral families (reviewed in refs. ^5–7^). APOBEC3G (A3G), in particular, is a powerful restriction factor of retroviruses that blocks viral replication by hypermutation of viral complementary DNA and inhibition of reverse transcription^1–3^. A3G is packaged into retroviral capsids through interactions with viral genomic RNA during assembly, exerting its antiviral activity inside the capsid where reverse transcription occurs during infection^8–12^. The lentiviral protein Vif inhibits A3G packaging into virions by targeting it for ubiquitin-mediated proteolysis, and through ubiquitin-independent mechanisms that are poorly understood^13–17^. On a long evolutionary timescale, A3G has undergone diversifying selection to escape antagonism by Vif whereas adaptations in Vif allow primate lentiviruses to expand their host range and adapt to host polymorphisms^18,19^. Repeated bouts of diversifying selection and adaptation are referred to as ‘molecular arms races’^20^. Adaptations in the Vif protein encoded by an SIV that infects red-capped mangabey monkeys (SIVrcm) to antagonize the hominid version of A3G enabled cross-species transmission of a lentivirus from monkeys to chimpanzees, which underlies the ancient origin of HIV-1 and the AIDS pandemic^21^. Although it is commonly assumed that sites of molecular arms races report on direct protein interactions, physical evidence of this interaction site to explain the mechanisms of how Vif promotes processive ubiquitination on A3G, and how mutations in Vif or A3G promote host escape and viral adaptation, remain critical and unresolved questions.

The A3 proteins are comprised of either one or two cytidine deaminase domains (CDAs) among which A3D, A3F and A3G (containing double-domains CDA1 and CDA2) and single-domain A3H inhibit replication of primate lentiviruses. To target A3 family members for ubiquitination and degradation, Vif hijacks a host Cullin-RING ubiquitin ligase (CRL) complex and a transcription cofactor core-binding factor beta (CBFβ)^16,22,23^. Costructures of Vif with full-length A3 family members have been a major challenge for the field due to poor solubility and difficulty in obtaining homogenous protein for structural studies. Accordingly, extensive effort has been devoted to the generation of variant A3 proteins that are soluble and amenable to structural studies^24–27^. For example, a 3.9 Å cryogenic electron microscopy (cryo-EM) structure of a solubility-optimized variant of A3F-CDA2 covalently fused to CBFβ bound to the Vif α/β domain showed an electrostatic interface required for viral infectivity^27^. However, the interaction of CDA2 of A3F with Vif and CBFβ is weak, and the importance of the tetramer comprising A3F-CDA2, the Vif α/β domain and CBFβ protomers for A3 antagonism is unclear^27–29^; moreover, the well-characterized evolutionary adaptations in A3 proteins in response to Vif occur in the CDA1 of A3G^19,30^, leaving substantial gaps in our knowledge of molecular mechanisms of Vif antagonism of A3 proteins and molecular arms races between them.

**Fig. 1:**
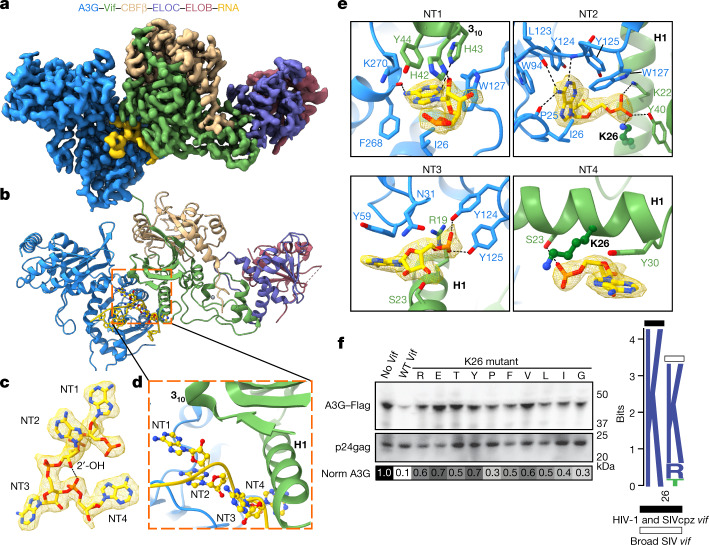
Structure of the VCBC ligase substrate receptor in complex with human A3G and RNA. **a**, Cryo-EM map for the A3G–RNA–VCBC monomer. **b**, Corresponding view of the refined coordinate model of the A3G–VCBC complex, highlighting the four-nucleotide core motif (ball-and-stick) between Vif and A3G. Here and throughout, the same colour coding for A3G, Vif, CBFβ, ELOB, ELOC and RNA is used as indicated. **c**, Composite density map for NT1–4 of RNA, with a hydrogen bond indicated between ribose 2′-OH on NT2 and phosphate on NT4. **d**, Ribbon diagram showing NT1–4 of RNA bridging helix 1 (H1) and 3_10_ helix turn of Vif with A3G. **e**, Close-up of protein–RNA interactions between Vif and A3G for each core nucleotide of RNA. **f**, Functional assessment of amino acid substitutions of residue K26 in HIV-1 Vif. Left, amino acid mutants at Vif residue K26 were assessed for their ability to prevent packaging of A3G into virions; top, virion incorporation of A3G; bottom, amount of virus (p24^gag^) in the corresponding virion preparation. Below is a greyscale heatmap of relative A3G incorporation normalized to p24^gag^ based on two replicate transfections (with the exception of K26Y), with the amount of A3G in the ‘No Vif’ control set to 1.0 (darkest shading). Controls were run on the same gel as the samples. For Source data, see Supplementary Fig. 1. Right, logo plot of amino acids found in the consensus of all HIV-1 clades, as well as SIVcpz (black bar) and all other SIV strains with equal distribution of each SIV (white bar). WT, wild type.

**Extended Data Fig. 1:**
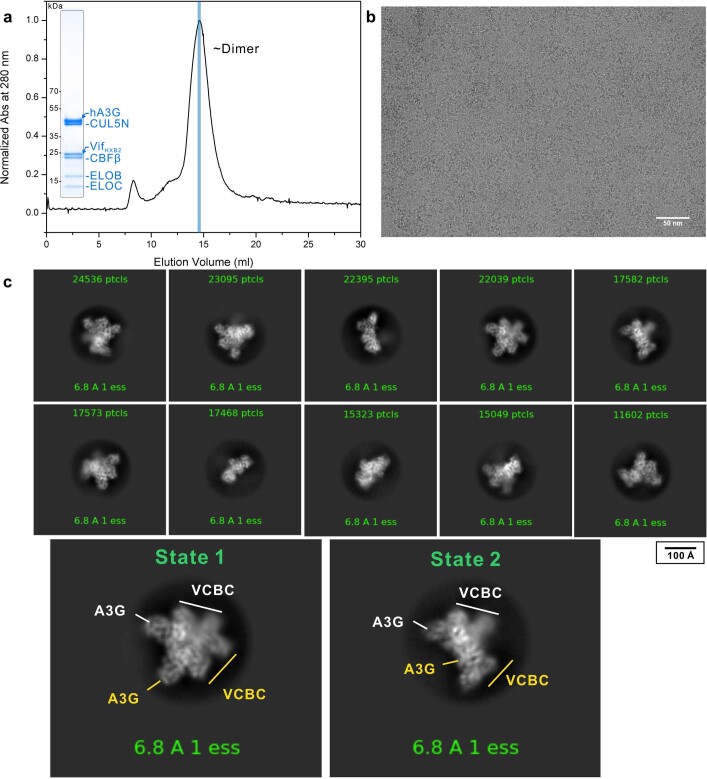
A3G–VCBC–CUL5N complex expression, purification, and characterization. **a**, Size exclusion chromatograph of purified A3G–VCBC–CUL5N complex along with a coomassie blue-stained SDS-PAGE (insert) of peak fraction (blue bar). The gel is representative of three independent experiments. For source gel data, see Supplementary Fig. 1b. **b**, A representative motion-corrected cryo-EM micrograph of purified complexes collected from peak fraction (blue bar in (**a**)) imaged on UltraAuFoil grid. Scale bar, 50 nm. **c**, Selected 2D class averages used for generating *ab initio* model in the first round of heterogeneous refinement (Extended Data Fig. 2a). Similar class averages were obtained from three independent preparations imaged on Quantifoil Gold grids. Scale bar, 100 Å. Shown at the bottom are expanded views of the fourth and fifth 2D classes with two copies of A3G-VCBC labeled in white and yellow.

**Extended Data Fig. 2:**
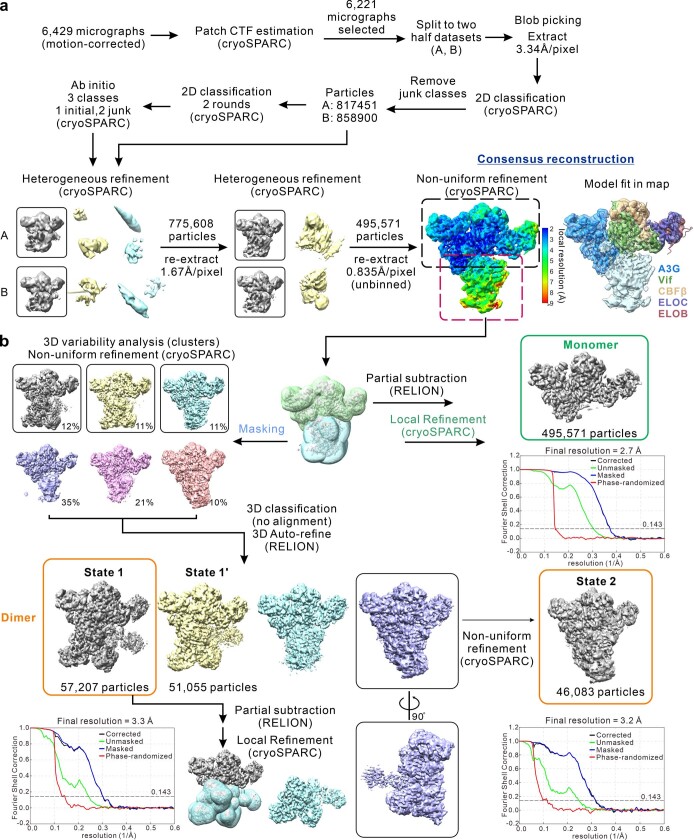
Workflow of cryo-EM image processing. Flowchart of pre-processing, classification, and refinement used to generate **a**, the consensus reconstruction map (monomer) and **b**, final maps (dimers) for model building. See Methods for details. Black boxes indicate the selected classes and corresponding particles used in the further refinement. The consensus reconstruction is colored according to the local resolution estimated by ResMap^73^. Shown on the right are the A3G and VCBC structures fit in the consensus map that is colored by subunits. Masks were used to determine different regions of volume for focused refinement and 3D variability analysis in cryoSPARC. Green and orange boxes indicate the final reconstruction for monomer and dimers, respectively; their corresponding Gold-Standard Fourier Shell Correlation (GSFSC) curves are shown at the bottom. The nominal resolution of the final map for monomer, State 1, and State 2 dimer is 2.7, 3.3, and 3.2 Å, respectively.

**Extended Data Fig. 3:**
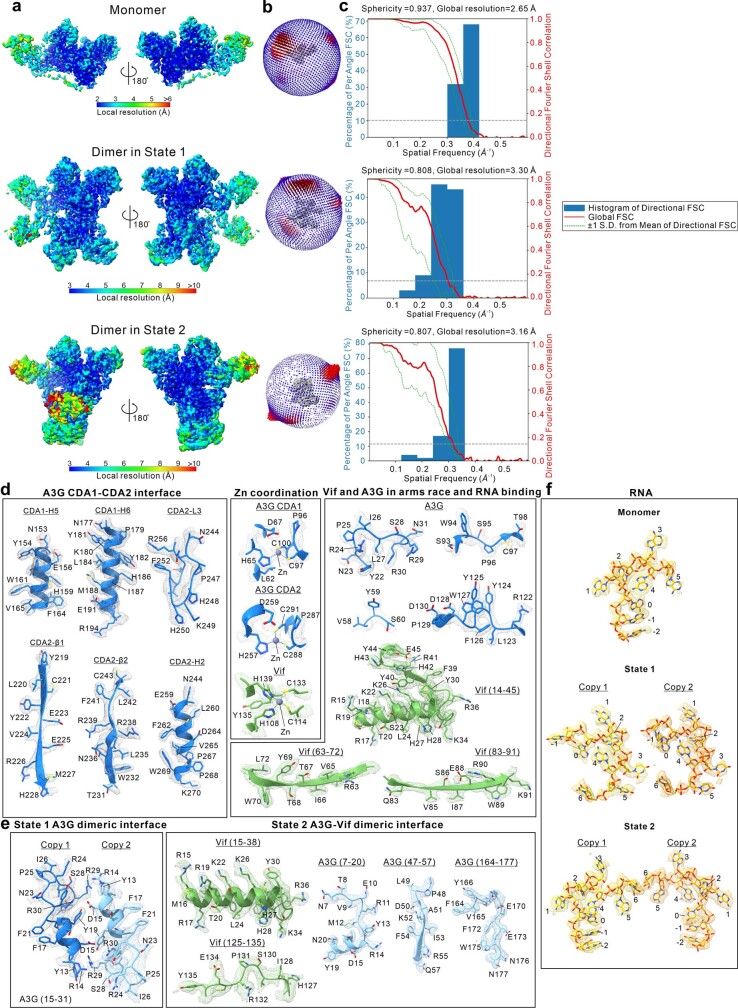
Cryo-EM map quality metrics. **a**, Front and back views of EM density maps colored by local resolution estimated by ResMap^73^. **b**, Euler angle distribution of the particles contributing to the final 3D reconstruction. **c**, Directional FSC plot for the reconstruction calculated by 3DFSC^74^. Shown are histograms of directional FSC values overlaid with global FSC (0.143 cutoff; red curve) and ±1 standard deviation from the mean of directional FSC (green curve). Sphericity of approximately 0.8 for State 1 and State 2 indicate mild resolution anisotropy in the reconstruction map, which might be caused by the slightly preferred orientation of particles shown in (**b**). **d-f**, Cryo-EM density and model fit for regions of interest of the reconstruction. Shown in mesh are EM maps with corresponding atomic models to demonstrate side chain density and map-model fit from various regions of the reconstructions: **d**, A3G CDA1-CDA2 (left), Zn^2+^ ion and residues within 5 Å of Zn^2+^ in the zinc finger domains of A3G and Vif (middle), arms race and RNA interfaces (right) from 2.7 Å monomer structure. **e**, Dimeric interface of State 1 and State 2. **f**, RNA in A3G–RNA–VCBC monomer, State 1 and State 2 dimer. See Supplementary Fig. 2–4 for map-model fit analysis by PHENIX^86^.

**Extended Data Fig. 4:**
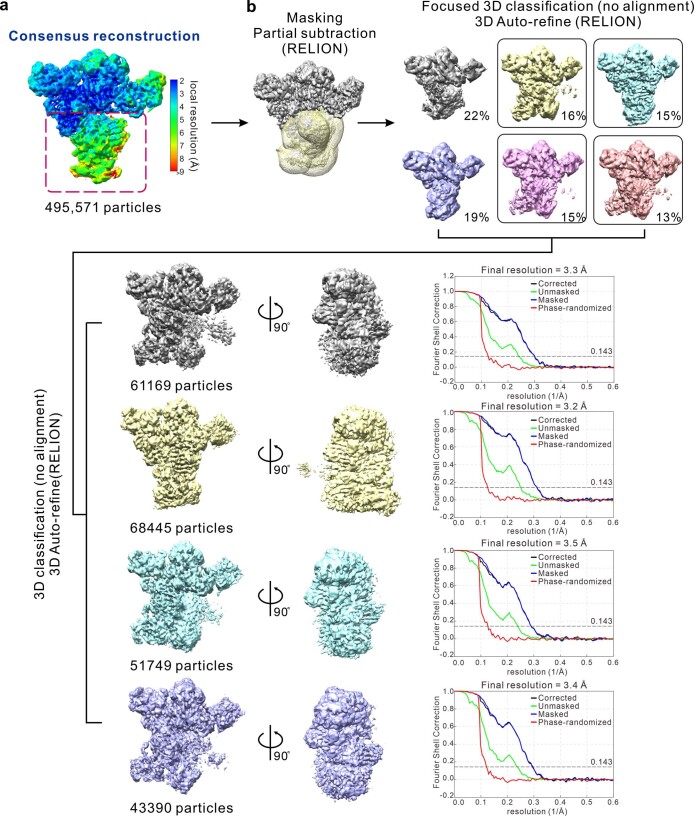
Image processing with focused classification and 3D refinement. **a**, The same consensus reconstruction map shown in Extended Data Fig. 2a. **b**, Focused classification with partial signal subtraction yielded 3D reconstruction maps with improved density quality of the bottom region and revealed two major conformational states as seen with 3D variability analysis (see Extended Data Fig. 2b). The mask used for focused classification is highlighted in yellow. Selected classes subjected to further processing are boxed in black. GSFSC plots are shown to the right of corresponding classes. See Methods for details.

Here, we solved the structure of wild-type human A3G bound to the substrate receptor module of CRL5 containing HIV-1 Vif, CBFβ, Elongin B and Elongin C (VCBC) using single-particle cryo-EM (Extended Data Table 1 and Extended Data Figs. 1–4). Two-dimensional (2D) classification indicates that the complex is a dimer of A3G–VCBC protomers (Extended Data Fig. 1c). Our highest-resolution map, generated by focused refinement around the monomer structure of A3G–VCBC, will be presented first, followed by the structures of the dimer (Methods, Fig. 1a,b and Extended Data Fig. 2).

## RNA bridges the Vif–A3G interaction

The crystal structure of VCBC and the AlphaFold2-predicted model of human A3G could be readily fit into a 2.7 Å-resolution map of the A3G–VCBC monomer (Fig. 1a,b)^31^. Unexpectedly, well-resolved density was observed for a single-stranded RNA (ssRNA) molecule sandwiched between A3G and Vif, most probably originating from the insect cells in which A3G–VCBC was co-expressed (Fig. 1a,b). Four nucleotides (named NT1–4) of ssRNA are wedged deeply between A3G and Vif, with RNA forming a sharp turn mediated by a hydrogen bond between ribose 2′ hydroxyl of NT2 and the phosphate backbone of NT4 (Fig. 1c,d). The majority of the interactions between A3G and Vif are mediated by CDA1, consistent with previous studies indicating that it binds RNA and is necessary and sufficient for binding to Vif^11,12,24,32–35^.

RNA binding has been implicated in the regulation of cytoplasmic localization, self-association and packaging of A3G into HIV-1 virus, which is essential for its antiviral activity^12,36–38^. The A3G CDA1 and CDA2 domains sandwich the RNA tetranucleotide at the interface formed by Vif helix 1 (residues 15–30), strand 2 (residues 39–41) and a 3_10_ helix (residues 42–46) (Fig. 1b–d). There is a division of labour between A3G and Vif in recognizing RNA. The bases of NT1–3 are bound to A3G, with aliphatic interactions, aromatic base stacking and hydrogen bonds to NT1 and NT2 typical of sequence-specific interactions (Fig. 1e). For example, the purine base of NT1 is buried in a junction formed by CDA1 (I26 and W127), CDA2 (K270) of A3G and Vif (H42, H43 and Y44), forming a hydrogen bond with the main-chain carbonyl of F268 on CDA2 (Fig. 1e). These interactions may explain why Vif residues lining the surface of the 3_10_ helix are important for A3G degradation and viral infectivity^35,39–44^. The purine base of NT2 is buried in a hydrophobic pocket formed by A3G residues (I26, W94, Y124, Y125 and W127) interacting with Y125 by T-stacking; NT2 also forms a hydrogen bond with the main-chain amide of Y125 and the carbonyl of P25 and L123 (Fig. 1e). Based on hydrogen bonding patterns, NT1 and NT2 are probably adenine. Sequence-specific interactions of NT1 and NT2 with A3G are consistent with the enrichment of purine-rich motifs that interact with A3G in cells and virions of *vif-*deficient HIV-1 (ref. ^45^). Both this result and our structure suggest that A3G bound to purine-rich RNA is the substrate of the Vif E3 ligase.

In contrast to sequence-specific interactions with purine NT1 and NT2 with A3G, NT3 and NT4 are stabilized by aromatic stacking interactions with Y59 of A3G and Y30 of Vif, respectively (Fig. 1e). A composite binding site for the RNA backbone is formed by Vif and A3G. Buried phosphates of NT2 and NT3 are stabilized by hydrogen bonds and salt bridges with Vif (residues Y40, K22 and K26) and A3G (Y124 and Y125), respectively (Fig. 1e). Almost all of the key contacts in the A3G–RNA–VCBC monomer interface have been mutated in previous genetic studies and result in a loss of Vif function (Supplementary Tables 1 and 2), which validates their importance in our structure.

**Extended Data Fig. 5:**
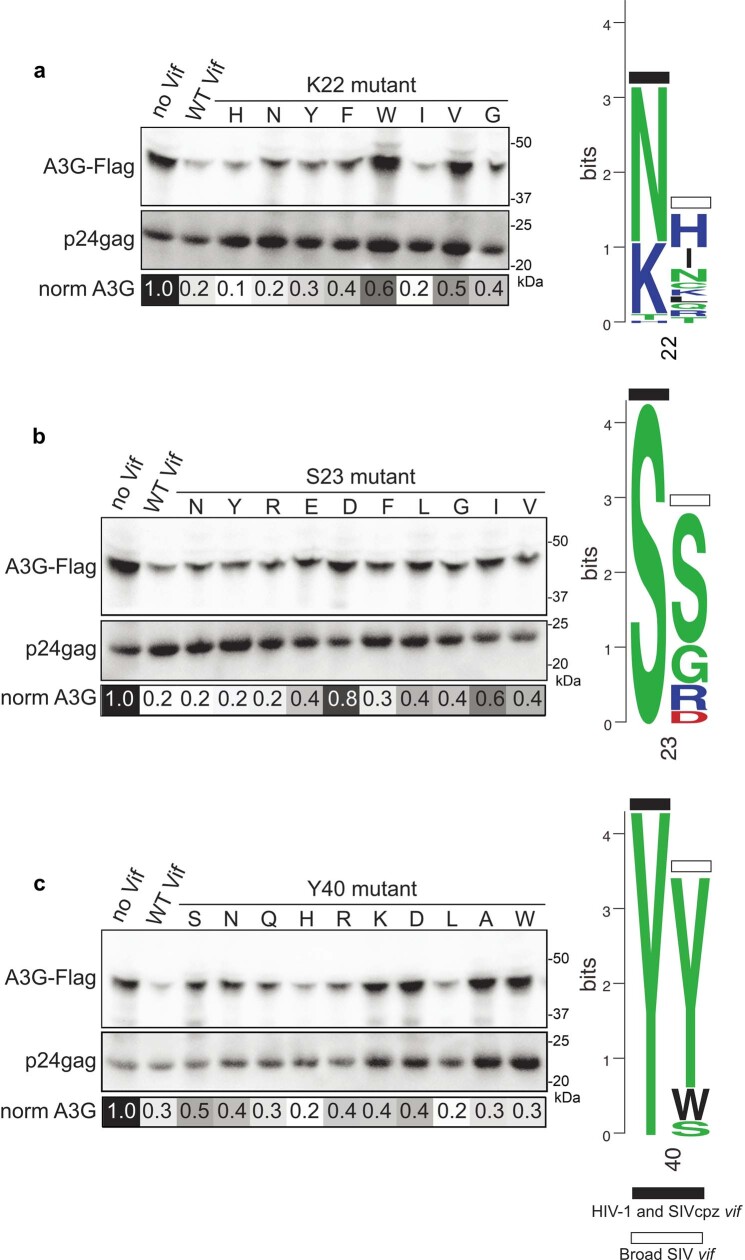
Functional and evolutionary assessment of Vif residues involved in RNA binding. Left: Amino acid mutants at Vif residue **a**, K22, **b**, S23, and **c**, Y40 were assessed for their ability to prevent packaging of A3G into virions. Top Western blot in each panel shows the virion incorporation of A3G, while the bottom Western blot in each panel shows the amount of virus (p24^gag^) in the corresponding virion preparation. Below each panel is a greyscale heatmap of the relative A3G incorporation normalized to p24^gag^ based on two replicate transfections (with the exception of S23Y and S23D) with the amount of A3G in the “No Vif” control set to 1.0 (darkest color). Controls were run on the same gel as the samples. For source data, see Supplementary Fig. 1a. Right of each panel: Logo plot of amino acids found in the consensus of all HIV-1 clades as well as SIVcpz (black bar), and all other SIV strains using equal distribution of each SIV (white bar).

Because amino acids at positions 22, 23, 26 and 40 of Vif make exclusive interactions with RNA, we substituted them with different classes of amino acid to test the role of RNA in Vif-mediated antagonism of A3G (Fig. 1e,f). Substitution of K26 was not tolerated, supporting its role in coordination of multiple interactions with the phosphate backbone of NT2 and NT4. By contrast, K22 and S23 were tolerant of polar amino acids but refractory to nearly all aromatic and aliphatic substitutions, consistent with their role in coordinating the phosphate backbone of NT2 and NT4 (Extended Data Fig. 5). The partial loss of function of Vif substitutions at Y40 may reflect the dual role of this residue, which interacts with RNA (NT2) and the 3_10_ helix of Vif. These functional results are largely reflected in the evolutionary constraints on Vif in HIV-1 and SIV sequences. For example, there is perfect conservation at residue 26 in HIV-1 and SIVcpz sequences whereas the only amino acids represented at position 22 are asparagine, lysine and threonine (Fig 1f and Extended Data Fig. 5a). These positions are also enriched with polar (residue 40) or charged (residues 22 and 26) amino acids in more divergent SIV Vif sequences, suggesting that the binding mode for A3G, RNA and Vif is deeply conserved (Extended Data Fig. 5). These results suggest that interaction of Vif with RNA is required for the antagonism of A3G. We conclude that RNA functions as a ‘molecular glue’ to stabilize Vif–A3G interactions, much like hormones or small molecules act to recruit substrates to cellular ubiquitin E3 ligase complexes^46,47^.

## Evolution of the Vif–A3G interface

**Fig. 2:**
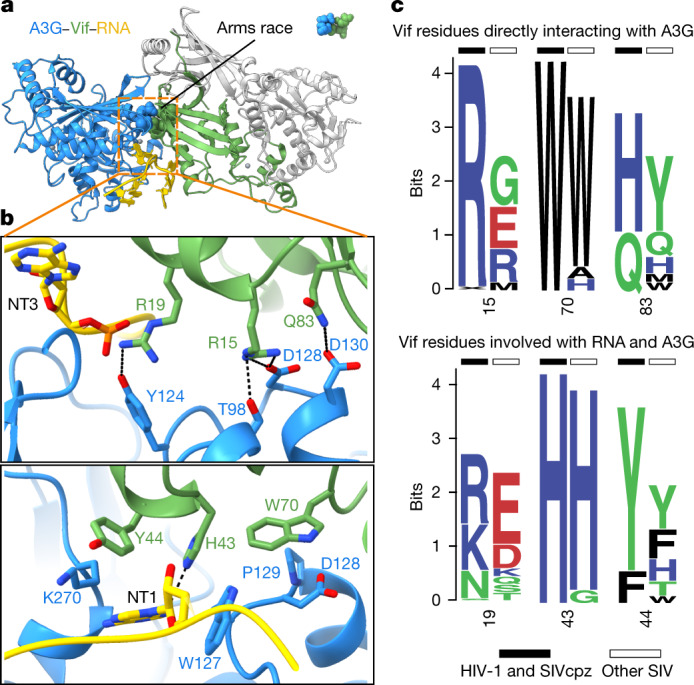
Interplay between the molecular arms race and RNA interfaces of Vif–A3G. **a**, Ribbon diagram showing position of molecular arms race interface (spheres) relative to the RNA interface (sticks). CBFβ, ELOB and ELOC are coloured grey. **b**, Close-up of molecular arms race interface (top) and residues that contribute to Vif–A3G binding and in contact with RNA (bottom). Residues D128 and D130 of A3G are under diversifying selection; residue Q83 is an adaptation that allowed SIVrcm Vif to neutralize hominid primate A3G and thus enable cross-species transmission. **c**, Logo plots of natural sequence variation in Vif residues that line the molecular arms race (top) and Vif–A3G–RNA interface (bottom). HIV-1 and SIVcpz sequences (black bars) are the consensus of all HIV-1 clades as well as SIVcpz, and SIV sequences (white bars) are all other SIV strains using equal distribution of each SIV.

**Extended Data Fig. 6:**
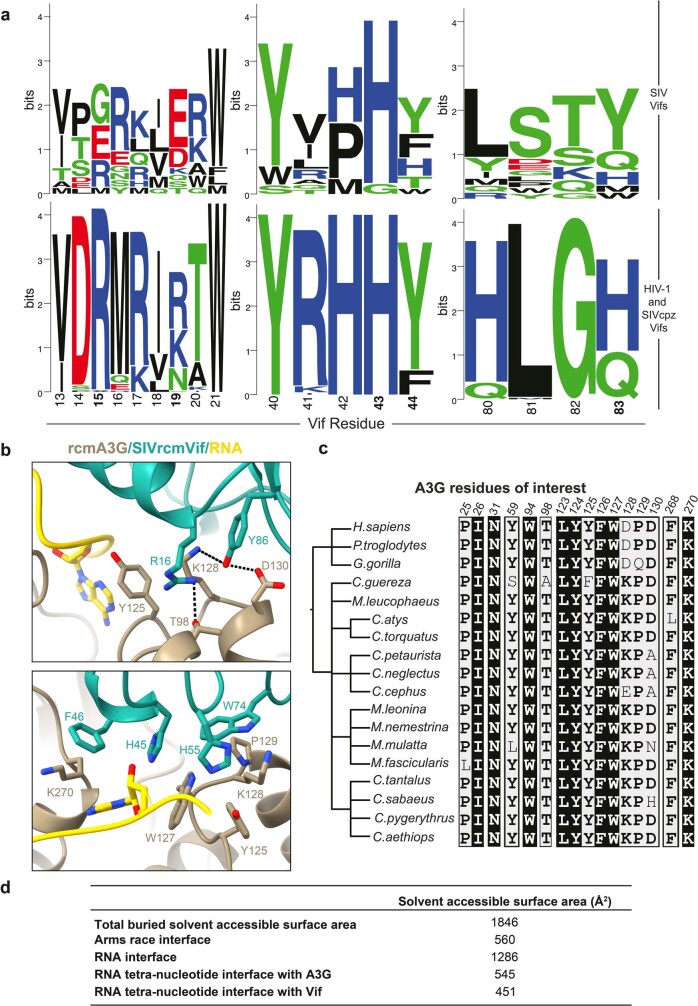
Characterization of arms race interface. **a**, Logo plots showing sequence variation of Vif residue that interacts with A3G for HIV-1 and SIVcpz (bottom) and other SIVs (top). Data is similar to that of Fig. 2c except that neighboring residues are also shown for context. **b**, A comparative model of rcmA3G–SIVrcm Vif–CBFβ was built with the hA3G-VCBC monomer structure as a template. Dashed lines represent the hydrogen bond network involved in the arms race interface (top). Residue K128 of rcmA3G interacts with Y86 of SIVrcm Vif, the primary determinant of Vif adaption to counteract hominid A3G^50^. Residue F46 and W74 of SIVrcm Vif previously reported to be critical for rcmA3G neutralization engage in extensive hydrophobic interactions with rcmA3G in the model (bottom). Note amino acids 16 and 86 of SIVrcm Vif correspond to amino acids 15 and 83, respectively, in HIV-1 Vif. **c**, Sequence alignment of A3G residues that contact RNA or Vif from Old World Monkeys and hominids. Fully conserved residues are highlighted with white text on black background. **d**, Buried solvent accessible surface area for A3G–RNA–VCBC monomer structure.

The identity of residues 128 and 130 in A3G has previously been shown to determine the species specificity of the adaptation of Vif to a new host species (reviewed in refs. ^30,48^). Here we call this interface with Vif the ‘arms race interface’, which has undergone diversifying selection during primate evolution^19,21,49^. The arms race interface is comprised exclusively of protein interactions between A3G and Vif and is adjacent to the RNA interface (Fig. 2a). Residues D128 and D130 of A3G are buried deep in the arms race interface, forming a network of hydrogen bonds with R15 and Q83 of Vif, respectively (Fig. 2b). Q83 of Vif was previously shown to be essential for the Vif adaptation that allowed cross-species transmission from SIVrcm to chimpanzee^50^ (Fig. 2b). Furthermore, W70 of Vif interacts closely with W127, D128 and P129 through hydrophobic interactions. This intimate network of contacts explains why lysine substitution at position 128 of A3G, observed in Old World monkeys, is unable to be counteracted by SIVcpz and HIV-1 Vif: it does not contain a hydrogen bond acceptor, nor the charge or shape complementarity to interact with R15 of Vif^49,51^ (Fig. 2b). R15 and W70 are conserved in all HIV-1 and SIVcpz Vif sequences, consistent with their functioning as lynchpins of the HIV-1 Vif interaction with human A3G (Fig. 2c). However, as predicted from an evolutionarily dynamic interface, the Vif sequences from Old World monkey SIV that must evolve to antagonize divergent host A3G residues in the arms race interface are themselves variable (Fig. 2c and Extended Data Fig. 6a)

Because the Vif gene that gave rise to SIVcpz, and ultimately to HIV-1, is derived from SIVrcm, we asked how the interaction between rcmA3G and Vif at the arms race interface may have evolved^21^. Comparative modelling of the rcmA3G–Vif complex based on our structure indicates that K128 of A3G directly interacts with Y86 of SIVrcm Vif (equivalent to residue 83 in HIV-1 Vif), suggesting that adaptation of Vif to the positively selected residue 128 entailed a remodelling of interactions occurring at the arms race interface (Extended Data Fig. 6b). We suggest that structural plasticity in Vif enabled amino acid substitutions, such as those occurring at position 86, to neutralize A3G and enable cross-species transmission of SIV from red-capped mangabeys to chimpanzees.

In contrast to the arms race interface, the interface between A3G and Vif that is bridged by RNA is well conserved because residues in the purine-binding pocket of A3G that contact RNA, such as the L7 loop (Y124–W127), are required for restriction in the absence of Vif (NT2; Fig. 1e, Extended Data Fig. 6c and Supplementary Table 2)^12^. We conclude that Vif binds A3G/RNA in a manner that limits A3G escape over long evolutionary timescales by engaging an essential surface required for antiviral function, explaining why genetic signatures of diversifying selection and adaptation are constrained to the direct protein interactions observed at the molecular arms race interface.

## Vif-mediated ubiquitination of A3G

**Fig. 3:**
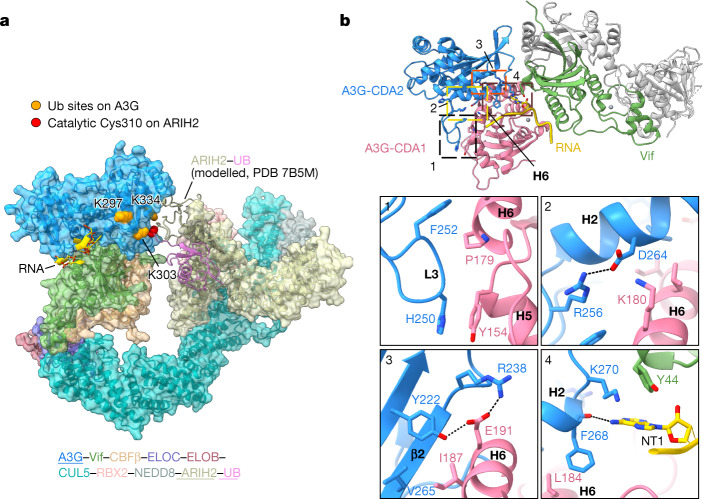
Vif orients acceptor lysine residues on CDA2 of A3G for ubiquitin transfer. **a**, Comparative model of A3G/RNA in complex with Vif CRL5 E3 ligase bound to coenzyme ARIH2 that transfers the first ubiquitin (Ub) to CDA2 of A3G. Lysine residues identified as A3G ubiquitination sites by mass spectrometry and required for Vif-mediated degradation of A3G are coloured orange^53–55^; the catalytic Cys310 of ARIH2 is coloured red. **b**, Overview of interactions that stabilize the relative orientation of CDA domains in A3G. Helix 6 (H6), previously shown to be important in A3G dimerization, is labelled^26^. Bottom panels show close-up of interactions within A3G CDA domains, and between A3G, RNA and Vif.

Cullin-RING E3 ligases conjugate ubiquitin onto substrates by orienting acceptor lysines into a ‘ubiquitination zone’ that is accessible by coenzymes^52^. To determine whether the A3G–RNA–VCBC module is compatible with ubiquitination by CRL5, we used comparative modelling. Lysine residues of A3G that are required for Vif-mediated ubiquitination and subsequent degradation are located within CDA2 of A3G, which is oriented towards the ARIH2 coenzyme of CRL5 that installs the first ubiquitin, allowing extension of K48-linked ubiquitin chains by a ubiquitin-conjugating enzyme^53–55^ (Fig. 3a). The orientation of CDA2 is determined by intramolecular interactions both in A3G and with Vif and RNA. Within A3G, helix 6 (residues 178–193) of CDA1 forms interactions with CDA2 that fix domain orientations (Fig. 3b) through a salt bridge (K180 with D264) and a series of hydrogen bonds (E191 with Y222, E191 with R238). Aromatic interactions between helix 5 of CDA1 (Y154) and the L3 loop in CDA2 (H250), as well as several hydrophobic contacts between both domains, stabilize CDA domain orientations (Fig. 3b). Interactions between A3G, Vif and RNA may fix the orientation of CDA2 in the three-way interface between K270, Y44 and NT1, respectively, suggesting that RNA not only acts as a molecular glue, but that it may also orient A3G CDA2 acceptor lysines for ubiquitin transfer (Fig. 3b).

Most of the intramolecular interactions in A3G are consistent with the AlphaFold2 structure of human monomeric A3G, but not with previous crystal structures of A3G containing solubility-enhancing mutations or rhesus macaque A3G, which exist in either monomer or self-associated forms^25,26^. Notably, in the crystal structure of rhesus macaque A3G, helix 6, which was proposed to promote self-association, is buried when human A3G is bound by VCBC^26^ (Fig. 3b). This observation indicates that VCBC binds A3G in a manner that inhibits its self-association.

## Conclusions and implications

Vif and A3G are a paradigmatic example of a host–pathogen molecular arms race. DNA sequence analyses of primate genomes and functional studies show two positions in A3G that undergo diversifying selection allowing escape from antagonists such as Vif^18,19^. Nevertheless, how Vif binds A3G sufficiently tightly to antagonize A3G remained unclear. We discovered that RNA acts like a molecular glue to promote a high-affinity interaction, because it increases the buried surface area of the Vif–A3G complex and is required for viral infectivity. Our structural studies show a small surface of protein–protein interactions between Vif and A3G that determines cross-species transmissions of primate lentiviruses, as well as the viral adaptations in Vif underlying the origin of HIV-1. Although the buried surface area of the arms race interface is twofold smaller than the RNA interface, it acts as a hot spot controlling the fate of viral infection (Extended Data Fig. 6d).

Previous biochemical and structural studies indicate that A3F-CDA2 makes transient interactions with Vif and CBFβ (refs. ^27–29^). Our work on wild-type A3G suggests both CDA domains and RNA make stable interactions with Vif without contacting CBFβ. In the former structural study, covalent fusion of A3F-CDA2 and CBFβ was used to increase the occupancy of A3F bound to Vif whereas, in our study, RNA achieves this role by acting as a molecular glue. It is well established that different A3 family members engage surface-exposed residues of Vif that are genetically separable^4^. We suggest that these surfaces may be bridged by cellular cofactors as described for A3G. An alternative, but not mutually exclusive, possibility is that interactions with Vif are stabilized by bipartite interactions with tandem CDA domains of A3 proteins. Structural studies of Vif–A3 complexes purified after coexpression or native purification from eukaryotic cells will allow this question to be addressed in future studies.

A new model for Vif antagonism of A3G is built on previous functional studies and two key observations from our structure. First, lentiviral genomes are enriched in purines, and cross-linking immunoprecipitation sequencing studies on cells infected with *vif*-deficient HIV-1 indicate that A3G preferentially binds to purine-rich sequences present in noncoding RNA, messenger RNA and viral genomic RNA^45,56^. Our structure shows that A3G binds to a purine-rich tetranucleotide motif using residues (Y124–W127) that are essential for viral packaging in the absence of Vif^11,12^ (Fig. 1c,e). We propose that the substrate of the Vif E3 ligase is not A3G but rather a complex of A3G bound to purine-rich RNA, including purine-rich sequences found in the viral genome.

**Extended Data Fig. 7:**
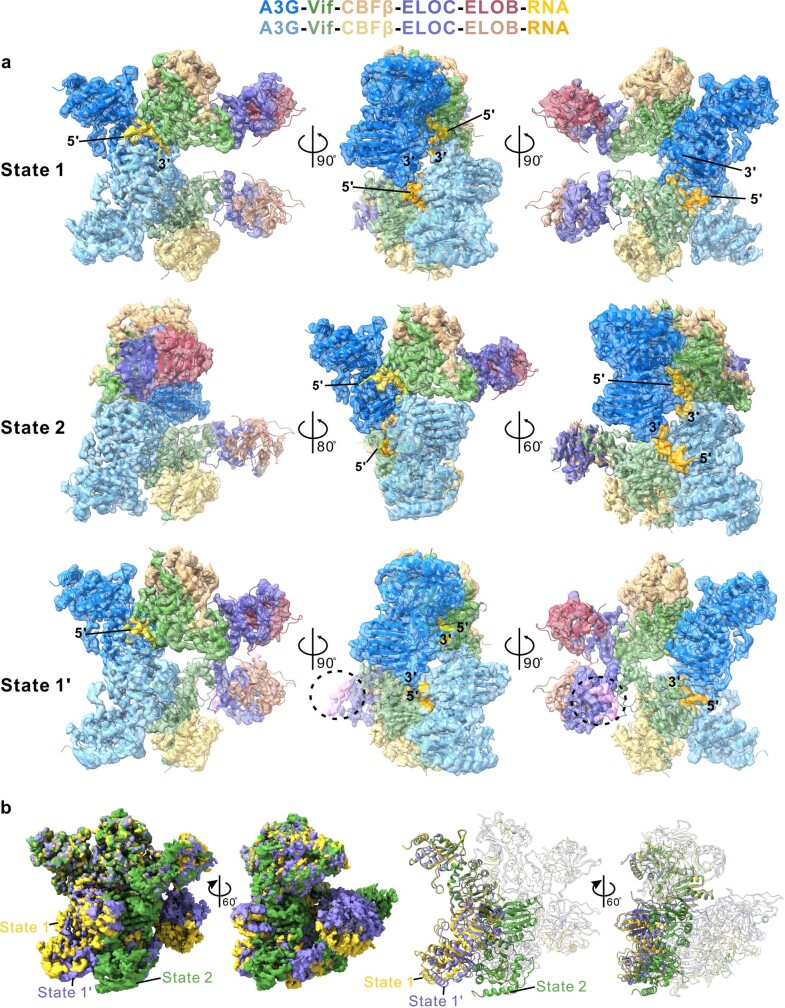
A3G-RNA-VCBC forms multiple discrete dimeric configurations. **a**, Cryo-EM maps for State 1 (top), State 2 (middle), and State 1′ (bottom) colored by subunit, showing three dimeric configurations of the A3G–RNA–VCBC complex (Extended Data Fig. 2b). Densities for single copies of RNA in State 1 and State 2 are clear for 8 and 9 nucleotides, respectively. In contrast, only 5 nucleotides were fit in the density map for State 1′ due to the moderate resolution at both ends of RNA. The 5′ and 3′ ends of single-stranded RNA are indicated. Extra weak density (pink) near ELOC corresponds to the expected position of CUL5N (denoted by black dashed circle) is observed for State 1′. State 1 and State 2 differ by a rigid-body motion with the second A3G–VCBC protomer rotated by 66° and translated by 28 Å relative to one another (Supplementary Video 1). **b**, A comparison of cryo-EM maps for State 1 (yellow), State 1′ (purple), and State 2 (green) in two orientations (left). Corresponding view of the structures for State 1, State 1′, and State 2, which are aligned by superposing A3G monomer (right). A3G is shown in solid ribbon while other proteins are in transparent for clarity. State 1′ has a dimeric configuration much similar to State 1, which are related by a 9° rotation and 4 Å translation. See Supplementary Discussion for the details.

**Extended Data Fig. 8:**
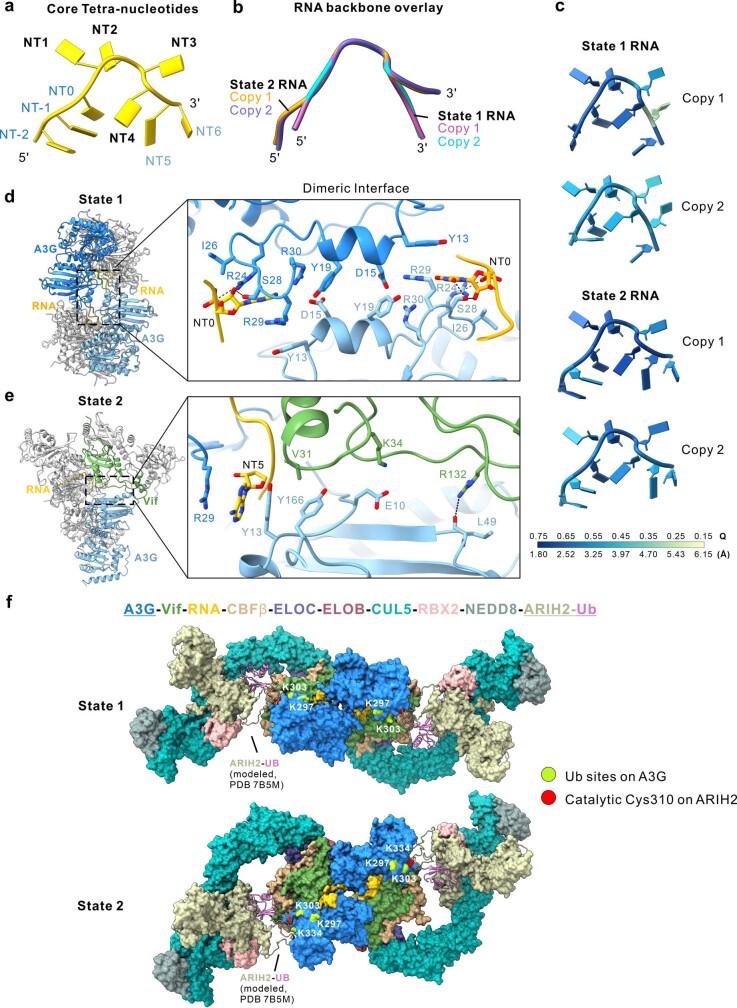
Dimeric interfaces in the different configurations of the A3G-RNA-VCBC complex. **a**, Schematic illustrating the annotation of RNA nucleotides used in this study. NT1-4 denotes the core tetra-nucleotides buried in the groove formed by A3G and Vif in the monomer; NT0 and NT5 are located near the dimeric interface for State 1 and State 2, respectively. **b**, Overlay of two copies of RNA backbone for State 1 and State 2. **c**, RNA models colored by per nucleotide Q-score^87^. The corresponding resolution estimated from each Q-score is indicated at the bottom of colored bar. The Q-score for State 1 and State 2 EM map is 0.54 and 0.56, respectively. **d–e**, Structure overview (left) and close-up (right) of the dimeric interface formed by (**d**) A3G and A3G for State 1 and (**e**) A3G and Vif for State 2. The buried solvent accessible surface area calculated by Chimera X^92^ is ~ 350 and ~ 480 Å^2^, respectively. Note that the RNA interface is in close proximity to the dimeric interface, suggesting RNA aids or dominates dimerization for both State 1 and State 2. See Supplementary Discussion for the details. **f**, Model of dimeric A3G-RNA-VCBC in complex with CUL5/RBX2 E3 ligase bound to ubiquitin-loaded ARIH2 (ARIH2~UB) for State 1 (top) and State 2 (bottom). States 1 and States 2 of A3G-RNA-VCBC are compatible with CUL5/RBX2 binding and ubiquitin transfer. The model was built by overlaying A3G-RNA-VCBC dimer structures determined in this study with published structure VCBC-CUL5_NTD_ (PDB code 4N9F), neddylated CUL5_CTD_–RBX2–ARIH2 (PDB code 7ONI), and a comparative model of ARIH2~UB (built based on a partial structure containing CUL1-RBX1-ARIH1-UB; PDB code 7B5M). Ubiquitin sites (K297, K303, and K334) on A3G are colored in lime, and catalytic Cys 310 on ARIH2 colored in red. See Supplementary Discussion for the details.

Second, in addition to RNA binding, A3G self-association is required for its packaging into virions^11^. Our structure indicates that Vif binding to A3G is mutually exclusive due to its ability to self-associate. Whereas A3G–VCBC forms dimers, within each dimeric assembly A3G forms little or no self-association (Extended Data Figs. 7 and 8 and Supplementary Discussion). This finding suggests that Vif binding to A3G has the capacity to block its packaging independent of ubiquitination activity, a mechanism that may potentiate repression of restriction.

**Fig. 4:**
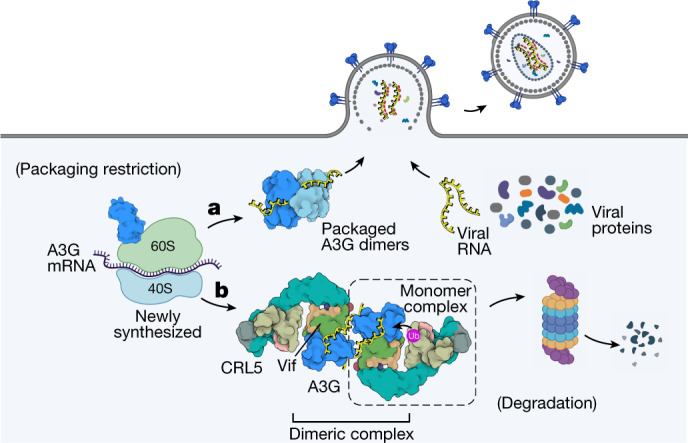
Schematic model of A3G inhibition by HIV-1 Vif. **a**,**b**, Packaging of A3G into HIV-1 virus requires A3G dimerization and its interaction with viral RNA (**a**); Vif neutralizes A3G early during its biosynthesis by binding RNA-bound A3G, inhibition of A3G dimerization and promotion of ubiquitin-mediated proteolysis (**b**). Created with BioRender.com.

In the absence of Vif, A3G self-associates onto viral genomic RNA and is packaged into viral particles for restriction (Fig. 4). We suggest that Vif antagonizes A3G early in its biosynthesis while it is a monomer in a specific complex with viral genomic RNA en route to viral packaging. Such an early intervention would ensure that Vif counteracts A3G in its most dangerous form for the virus, disrupting encapsidation and promoting polyubiquitination while bound to genomic RNA. Nucleotides that are 3′ to the primary Vif–A3G interaction site may template an additional copy of the Vif E3 ligase to cooperatively reinforce Vif–A3G interactions and ubiquitination (Fig. 4 and Extended Data Fig. 8f). This model is consistent with observations that newly synthesized, low-molecular-mass forms of A3G are packaged and most sensitive to Vif-mediated degradation, explaining ubiquitin-dependent and -independent functions of Vif and how the plasticity of molecular arms races can be enabled by a third party such as RNA.

In summary, A3G binding to Vif is not restricted to the evolutionary dynamic interface subject to diversifying selection and adaptation, contrary to popular models of molecular arms races, but rather also includes a conserved interface through RNA binding that helps position key residues necessary for viral antagonism of a host antiviral gene—a principle that may be adopted by other innate immune proteins and pathogen-encoded antagonists^57,58^.

## Methods

### Protein expression and purification

Full-length genes of HIV-1_HXB2_ Vif, CBFβ, ELOB, ELOC, human A3G with C-terminal Strep-tag and Cullin 5 with C-terminus truncated (1–386; abbreviated as CUL5N) were cloned into a single MacroBac vector, 11A (Addgene, no. 48294) using the restriction-ligation method as previously described^59^. A recombinant baculovirus encoding all six proteins was generated using the Bac-to-Bac baculovirus expression system (Thermo Fisher Scientific)^60^. A suspension of Sf9 insect cells was maintained in SF900 III SFM medium (unauthenticated, regularly tested for mycoplasma contamination; Thermo Fisher Scientific, no. 12659017) in a shaker (Innova 4430 incubator shaker) at 120 rpm and 27 °C. Serially diluted recombinant baculoviruses were added to 25 ml of Sf9 cells (2 × 10^6^ cells ml^–1^) grown in a polycarbonate Erlenmeyer flask (Corning), with protein expression evaluated by SDS–polyacrylamide gel electrophoresis (SDS–PAGE) and immunoblotting analysis to optimize the ratio of virus volume to cell volume. One litre of Sf9 insect cells (2 × 10^6^ cells ml^–1^) was infected with virus at a ratio determined from small-scale titration experiments, and cultured for 48 h before collecting by centrifugation (1,500*g*, 10 min). Cell pellets were washed with PBS and resuspended in fivefold the pellet volume of lysis buffer (50 mM HEPES, 50 mM NaCl, 5% glycerol, 1% Triton X-100, 5 mM MgCl_2_, 5 mM CaCl_2_, 1 mM Tris(2-carboxyethyl)phosphine (TCEP), mini cOmplete protease inhibitor cocktail (Sigma-Aldrich), 25 μg ml^–1^ DNase I (Sigma-Aldrich) and 50 μg ml^–1^ RNase A (Sigma-Aldrich), pH 8.0) and lysed by Dounce homogenizer. All purification steps were performed at 4 °C. Cell lysates were clarified by centrifugation at 17,000*g* (F15-8x50cy rotor) for 2 h. Supernatants were filtered (0.45 μm) and loaded onto a 5 ml StrepTrap HP column (GE Healthcare) pre-equilibrated in binding buffer (50 mM HEPES, 150 mM NaCl, 5% glycerol, 1 mM TCEP, pH 8.0). The column was washed with 15 column volumes (CV) of wash buffer (50 mM HEPES, 1.5 M NaCl, 5% glycerol, 1 mM TCEP, pH 8.0), followed by 5 CV of binding buffer. The protein was eluted in 6 CV of binding buffer supplemented with 5 mM d-desthiobiotin (Sigma-Aldrich). The eluate was dialysed overnight against 1 l of dialysis buffer (50 mM HEPES, 75 mM NaCl, 5% glycerol, 1 mM TCEP, pH 7.0). The sample was applied to a 5-ml HiTrap Heparin column (GE Healthcare) pre-equilibrated with dialysis buffer and eluted over a 0–100% linear gradient of elution buffer (50 mM HEPES, 1 M NaCl, 5% glycerol, 1 mM TCEP, pH 7.0). Fractions containing all six proteins were pooled, dialysed against 1 l of running buffer (30 mM HEPES, 150 mM NaCl, 5% glycerol, 1 mM TCEP) for at least 4 h, loaded onto a Superose 6 increase 10/300 GL column (GE Healthcare) pre-equilibrated with running buffer, and 0.3 ml fractions were collected. Size-exclusion chromatography indicates that the particle is a dimer in solution consisting of two copies of A3G–VCBC–CUL5N (roughly 330 kDa) (Extended Data Fig. 1a). The peak fraction was used directly for cryo-EM without further concentration (Extended Data Fig. 1a,b).

### Cryo-EM sample preparation and data acquisition

Purified complex (3.5 μl, 1.9 μΜ) was applied to glow-discharged UltraAuFoil 300 mesh R1.2/1.3 grids (Electron Microscopy Science), incubated for 15 s at 23 °C and 100% humidity, blotted with a blot force of 0 for 12 s then plunge-vitrified into liquid ethane using a FEI Vitrobot Mark IV (Thermo Fisher). A total of 6,429 super-resolution videos were collected at a nominal magnification of ×105,000 on a FEI Titan Krios microscope (Thermo Fisher), equipped with a K3 direct electron detector and BioQuantum energy filter (Gatan) and set to a slit width of 20 eV. Collection was performed semiautomatically using SerialEM at a dose rate of 8.0 e^–^ pixel^−1^ s^−1^ for a total dose of 68 e^–^ Å^–2^ over 118 frames^61^. Dose-fractionated image stacks were motion corrected, dose weighted and 2× binned to the physical pixel size of 0.835 Å by MotionCor2 in the package SCIPION^62,63^. A defocus range of −0.8 to −2.0 μm was applied.

### Image processing and 3D reconstruction

Initial processing of the resulting summed micrographs was performed in cryoSPARC v.3.0 (ref. ^64^) (Extended Data Figs. 1 and 2a and Extended Data Table 1). The contrast transfer function (CTF) of dose-weighted, motion-corrected micrographs was estimated by Patch CTF. Micrographs with CTF fit resolution poorer than 4 Å and excessive ice contamination were removed, resulting in a final total of 6,221 micrographs. Selected micrographs were split into two half datasets to speed up data processing. Approximately 2.3 million particles were picked using cryoSPARC circular blob, with a minimum and maximum particle diameter of 150 and 200 Å, respectively, and minimum separation distance between particles of 108 Å, extracted, and 4× binned (3.34 Å per pixel). After 2D classification of extracted particles, class averages without proteinaceous features were discarded. The remaining particles were subjected to two rounds of 2D classification, resulting in classes with clear structural features used to generate a good initial model (Extended Data Fig. 1c). Particles saved from the first round of 2D classification underwent iterative rounds of ab initio reconstruction and heterogeneous refinement using three reference maps (one good and two junk) (Extended Data Fig. 2a). Unbinned particles were re-extracted from the best reconstruction and refined with nonuniform refinement. The resulting three-dimensional (3D) map (designated as ‘consensus reconstruction’) features a well-resolved top body (Extended Data Fig. 2a, black-dashed box) and a bottom body of relatively poor resolution (Extended Data Fig. 2a, red-dashed box).

The top body of the consensus reconstruction map shows visible helical features in which we were able to fit the crystal structure of HIV-1 VCBC (PDB: 4N9F) and the AlphaFold 2-predicted human A3G monomeric structure (AF2: Q9HC16)^31,65^ (Extended Data Fig. 2a, black-dashed box). To improve the local density of the top body, particle subtraction and focused refinement were applied^66^. Using Chimera^67^ and RELION^68^, a mask was applied to subtract the signal of the bottom body from particle images. These signal-subtracted particles were then reimported into cryoSPARC and subjected to local refinement using a soft mask around the top body. The resulting focused, refined map is termed a ‘monomer’ density map because it accommodates the A3G–VCBC monomer structure well, at a nominal resolution of 2.7 Å (Extended Data Fig. 2b, green box). The local resolution of the density map is variable (Fig. 1a,b and Extended Data Fig. 3). A3G, Vif and CBFβ have well-resolved side-chain density, allowing reliable model building and refinement (Extended Data Fig. 3). The resolution of ELOC and ELOB is sufficient for backbone tracing, but most density for sidechains was absent. CUL5N was present in the preparation but not in our final maps, presumably due to the dynamic features of VCBC–CUL5N or dissociation during freezing^15,29^.

To address the conformational heterogeneity of the bottom body (Extended Data Fig. 2a, red-dashed box), the 495,571 particles from this consensus refinement were subjected to 3D variability analysis in cryoSPARC^69^ (Extended Data Fig. 2b). Particles were reclassified into six clusters using three principal components with a soft mask enclosing the bottom body and filter resolution set to 8 Å, followed by nonuniform refinement in cryoSPARC. Four out of six selected classes were then imported into RELION and 3D classification was performed without alignment using a *T* value of 4 to sort remaining low-quality particles. Classes showing strong density for the bottom body were further processed with 3D autorefine in RELION. The reconstructions showed improved densities in the bottom region, in which three distinct conformational states could be identified at a nominal resolution of 3.3 Å (state 1, 57,207 particles), 3.5 Å (state 1′, 51,055 particles) and 3.46 Å (state 2, 48,310 particles), respectively. The two highest-quality maps (states 1 and 2) were refined further, and the resulting maps allowed fitting of an additional copy of the A3G–VCBC complex (Extended Data Figs. 2b and 7).

Local refinement of state 1 without the top body was performed later in cryoSPARC, leading to a reconstruction with more complete density in this region. The state 2 reconstruction was subjected to a further round of nonuniform refinement in cryoSPARC to improve anisotropy, reaching a nominal resolution of 3.16 Å (Extended Data Fig. 2b). The discrete conformational states of the A3G-VCBC dimer observed with 3D variability analysis were also validated by focused classification in RELION^66^ (Extended Data Fig. 4). The density of the top body was subtracted from the particle images of the consensus reconstruction (Extended Data Fig. 4a; red-dashed box highlights the density to be retained). These signal-subtracted particle images were classified into six classes without alignment, using a soft mask focused on the bottom body (Extended Data Fig. 4b). Following refinement by 3D autorefine, four out of six selected classes were subjected to 3D classification skipping alignment and 3D autorefinement in RELION. The resulting 3D classes are consistent with those generated by 3D variability analysis in cryoSPARC, differing only in the density levels of the bottom copy. We used the reconstruction maps generated from 3D variability analysis to build the model, owing to their stronger overall density. Postprocessing of the final reconstruction was performed in RELION for estimation of global resolution using a Fourier shell cutoff (FSC) of 0.143 (ref. ^70^). The maps were sharpened with DeepEMhancer^71^ and improved by density modification without a model applied in PHENIX^72^ for map interpretation and model building. Local resolution estimation was done by ResMap^73^. Directional resolution was assessed using the 3DFSC server^74^. Format conversion between software was carried out with PyEM^75^.

### Model building and refinement

A comparative model of HIV-1 Vif_HXB2_–CBFβ was built with MODELLER^76,77^ using the X-ray structure of HIV-1 Vif_NL4-3_CBC–CUL5_NTD_ (PDB code 4N9F) as a template^31^. The atomic model for hA3G–V_HXB2_CBC was generated by fitting separate models of human A3G (AF2 code Q9HC16), the aforementioned comparative model of Vif_HXB2_–CBFβ and ELOB/C from the SIVrcm VCBC structure (PDB code 6P59) into the monomer density map using UCSF Chimera^50,65,67^. This starting model was manually rebuilt in Coot^78,79^ and adjusted in ISOLDE^80^ to improve local fitting. The model was then real-space refined in PHENIX^81,82^. The refined structure obtained from the monomer density map was used as a template for model building of two copies of A3G–VCBC into the EM maps for states 1, 1′ and 2. The same model-building and refinement procedure were performed for states 1 and 2 because of their higher overall resolution. Most residues buried at the A3G–Vif and A3G–A3G interface in the monomer and dimers showed clear density for the side chains, except for Vif residues 117–154 in the bottom copy of state 2 (Extended Data Fig. 3d,e). The weak density in this region precluded precise atomic modelling, and thus the A3G–Vif dimeric interface for state 2 is interpretable on one side only.

After model building the A3G–VCBC proteins, we observed unaccounted-for density sandwiched between A3G and Vif in the EM maps for state 1 and 2 dimers, which was annotated as oligonucleotides by Haruspex, a convolutional neural network trained to detect DNA/RNA versus protein in density maps^83^. The copurified RNA probably originated from the insect cells where A3G–VCBC was co-expressed. The continuous phosphate backbone density could be traced and well fit with ssRNA. The densities of copurified RNA, especially those sandwiched between A3G and Vif, are clearly resolved, showing features that allow distinguishing of purines and pyrimidines (Fig. 1a–c and Extended Data Fig. 3f). Guided by the EM density of RNA, a dummy sequence containing either adenine or uridine was manually built into the EM map in Coot. RNA geometry was improved by ERRASER^84^. The complete model, including RNA, was assessed using MolProbity^85^ and optimized after iterative refinement in Coot and PHENIX. Model–map fit was evaluated by correlation coefficient in PHENIX and *Q*-score analysis^86,87^. Protein–RNA interactions were detected by BINANA^88^ and protein-only interactions were analysed by PLIP^89^ and Ligplot^90^ with default settings, except that the hydrogen bond distance cutoff was set to 3.5 Å. Orientation and displacement between state 1 and 2 structures were determined using PyMOL^91^. The morph video was generated in UCSF Chimera X. Figures 1a–e, 2a,b, 3, as well as Extended Data Figs. 3, 7, 8, were created using Chimera X^92^. Extended Data Figs. 2, 4 were generated using Chimera^67^ and Chimera X^92^. Figure 4 was created using Chimera X^92^ and BioRender.com. Model statistics are summarized in Extended Data Table 1.

### Comparative modelling of rcmA3G–Vif–CBFβ complex

Because the atomic structure of the rcmA3G–Vif complex is not available, we built a comparative model of rcmA3G bound to Vif–CBFβ using MODELLER v.10.1 (refs. ^76,77^). Sequence identity between the template and model was 79, 40 and 100% for A3G, Vif and CBFβ, respectively. A3G residues required for RNA binding are sequence conserved among hominids and Old World monkeys (Extended Data Fig. 6c). The coordinates of RNA and Zn^+2^ ions were transferred from the templates to the generated model. After computing around 600 models, we used hierarchical clustering and DOPE scoring to obtain the top-scoring cluster^93^. The precision of this cluster is 1.4 Å; model precision is defined as the variability among the structural models. The best-scoring model was used for further analysis and is shown in Extended Data Fig. 6b.

### Comparative modelling of A3G–Vif–CBFβ–CRL5–NEDD8–ARIH2–Ub

Using MODELLER, we computed a comparative model of the full A3G–Vif–CBFβ in complex with neddylated CRL5 and coenzyme ARIH2. Templates included the A3G–Vif–CBFβ–ELOB/C structure presented here, the Vif–CBFβ–CUL5_NTD_–ELOB/C pentameric complex (PDB code 4N9F)^31^, the neddylated CUL5_CTD_–RBX2–ARIH2 tetrameric complex (PDB code 7ONI)^94^ and a partial structure containing CUL1–RBX1–Ub–ARIH1 (PDB code 7B5M)^52^. We computed roughly 100 models, which were clustered and evaluated using DOPE scoring^93^. The precision of the top-scoring cluster is 1.6 Å. The best-scoring model was used for further analysis and is shown in Fig. 3a.

### Assay of *vif* mutants for A3G degradation

We generated a library of variants at positions 22, 23, 26 and 40 using degenerate oligonucleotide mutagenesis in the HIV-1 LAI *vif* gene. Individual colonies were sequenced and ligated into a lentiviral vector. Human A3G, flanked by a C-terminal 3XFLAG epitope tag in the pcDNA4/TO vector backbone (Thermo Fisher, no. V102020), was transfected into HEK293T cells (ATCC CRL-3216, unauthenticated, regularly tested for mycoplasma contamination) plated in six-well dishes at a density of 1.5 × 10^5^ cells ml^–1^. The amount of A3G packaged into virions was assayed by cotransfection of 1,000 ng of Vif vector, 200 ng of A3G-3XFlag and 500 ng of psPAX2 for gag/pol production with TransIT-LT1 transfection reagent (Mirus, no. MIR2304) at a reagent to plasmid DNA ratio of 3:1. Two days post transfection, 1 ml of the supernatant was filtered through a 0.2 μm syringe filter and virions were pelleted in an Eppindorf 5415R tabletop microcentrifuge for 1 h at 4 °C and maximum speed. The supernatant was aspirated off, and 25 μl of NuPAGE 4× loading dye (Invitrogen no. NP0007) was added to each sample. Samples were boiled for 10 min at 95 °C and loaded on an SDS–PAGE gel. Anti-FLAG (Sigma, no. F3164) and anti-p24^gag^ (NIH-ARP, no. 3537) antibodies were used for immunoblotting at a dilution of 1:5,000. Mouse IgG HRP-conjugated antibody (R&D systems, no. HAF007) was used to detect primary antibodies at a dilution of 1:5,000. Chemiluminescent signals from all immunoblots were imaged using the ChemiDocMP imaging system (Bio-Rad), and images were processed with ImageJ software to quantify the densitometry for each detected antibody band. Normalized A3G in virions was calculated by dividing the amount of A3G by that of p24gag and setting that number to 1.0 for the ‘No Vif’ control.

### Natural sequence variation analysis

For analysis of natural variation in Vif at interaction sites with RNA and A3G, a curated subtype reference alignment for HIV-1 and SIVcpz sequences was downloaded from the Los Alamos HIV database (https://www.hiv.lanl.gov/content/index). This alignment includes four representatives from each HIV-1 group M subtype and four from groups N, O and P, as well as 21 SIVcpz sequences from each of the *Pan troglodytes* subspecies (*troglodytes* and *schweinfurthii*) sampled from primary isolates comprehensively encompassing the geographic range. Consensus sequences of SIVasc, SIVdeb, SIVdrl, SIVlst, SIVgsn, SIVmac, SIVmus, SIVrcm, SIVsmm, SIVgrv, SIVver, SIVtan, SIVsun, SIVsab and SIVgor were generated from all available sequences in each respective SIV. These sequences were aligned using Clustal Omega, and logo plots were generated from these alignments with WebLogo^95^.

To generate A3G sequence alignments, sequences were downloaded from NCBI (accession nos. AGI04219.1, AAP85255.1, Q694C1.1, AGE34499.1, NP_001332845.1, XP_011887342.1, AGE34492.1, AGE34504.1, AGE34486.1, AGE34487.1, ANY26448.1, XP_011710628.1, AGX93019.1, XP_011710628.1, AEY75956.1, NP_001279005.2, AEY75955.1, Q7YR25.1). Sequences were aligned with Clustal Omega and visualized using ESPript 3 (ref. ^96^).

### Reporting summary

Further information on research design is available in the Nature Portfolio Reporting Summary linked to this article.

## Online content

Any methods, additional references, Nature Portfolio reporting summaries, source data, extended data, supplementary information, acknowledgements, peer review information; details of author contributions and competing interests; and statements of data and code availability are available at 10.1038/s41586-023-05779-1.

### Supplementary information

Supplementary Information Supplementary discussion, Figs. 1–4, Tables 1 and 2 and references. Supplementary discussion: this file discusses the structure of A3G-RNA-VCBC dimeric complexes, corresponding to Extended Data Figs. 1–4 and 7–8. Supplementary Fig. 1 contains uncropped source images for Fig. 1f and Extended Data Figs. 1a and 5. Supplementary Figs. 2–4 show the model–map fit for A3G-RNA-VCBC monomeric and dimeric complexes. Supplementary Table 1 summarizes Vif residues reportedly involved in RNA binding based on mutational analyses. Supplementary Table 2 summarizes A3G residues reportedly involved in RNA binding based on mutational analyses.

Reporting Summary

Supplementary Video 1 Comparison of dimeric complex structure in states 1 and 2. States 1 and 2 are related by rigid-body motion of A3G-RNA-VCBC protomers. A3G is highlighted in pink.

## Figures and Tables

**Extended Data Table 1:**
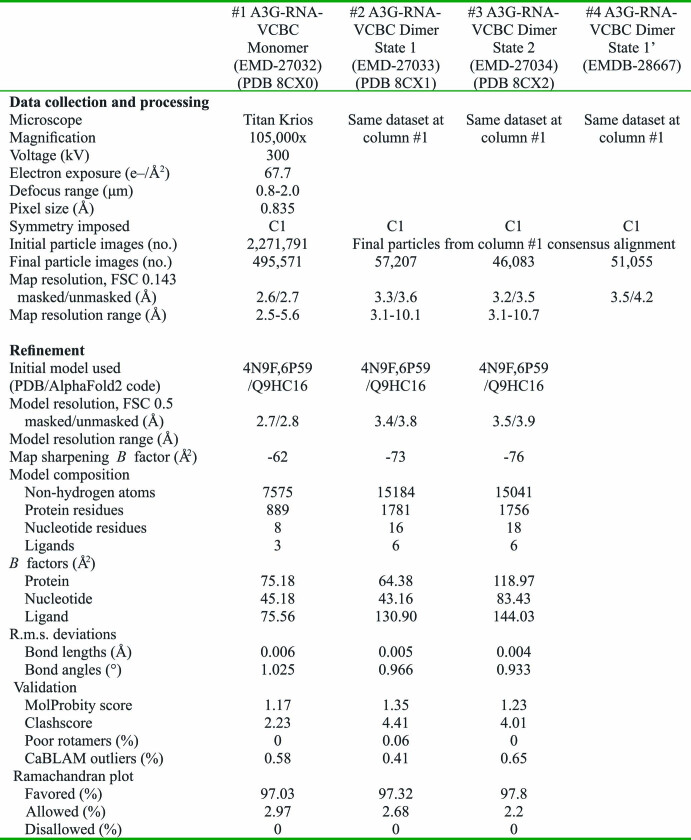
Cryo-EM data collection, refinement and validation statistics

## Data Availability

Cryo-EM maps and maps focused on specific regions used to guide model building are deposited in the Electron Microscopy Data Bank with accession codes EMD-27032 (A3G–RNA–VCBC monomer), EMD-27033 (A3G–RNA–VCBC dimer for state 1), EMD-27034 (A3G–RNA–VCBC dimer for state 2) and EMD-28667 (A3G–RNA–VCBC dimer for state 1’). The associated coordinate files are deposited in the Protein Data Bank with accession code 8CX0 (A3G–RNA–VCBC monomer), 8CX1 (A3G–RNA–VCBC for state 1) and 8CX2 (A3G–RNA–VCBC for state 2).
