# Supplementary material for: The structural basis for HIV-1 Vif antagonism of human APOBEC3G
Source: Nature. 2023 Feb 8;615(7953):728–33. doi: 10.1038/s41586-023-05779-1 (PMC10033410; doi:10.1038/s41586-023-05779-1)
Supplement: Supplementary file 1 — Supplementary discussion, Figs. 1–4, Tables 1 and 2 and references. Supplementary discussion: this file discusses the structure of A3G-RNA-VCBC dimeric complexes, corresponding to Extended Data Figs. 1–4 and 7–8. Supplementary Fig. 1 contains uncropped source images for Fig. 1f and Extended Data Figs. 1a and 5. Supplementary Figs. 2–4 show the model–map fit for A3G-RNA-VCBC monomeric and dimeric complexes. Supplementary Table 1 summarizes Vif residues reportedly involved in RNA binding based on mutational analyses. Supplementary Table 2 summarizes A3G residues reportedly involved in RNA binding based on mutational analyses. [file 41586_2023_5779_MOESM1_ESM.pdf]

nature portfolio

https://doi.org/10.1038/s41586-023-05779-1

Supplementary information

The structural basis for HIV-1 Vif antagonism of human APOBEC3G

In the format provided by the authors and unedited

SUPPLEMENTARY DISCUSSION

RNA strand swapping promotes multiple dimeric assemblies of A3G-RNA-VCBC

The A3G-VCBC monomer complex forms dimers in at least three different configurations, State 1, State 1’, and State 2 through interaction with two single-stranded RNA molecules (Extended Data Fig. 7a). The configuration of State 1’ is similar to State 1, but the density quality of nucleotides flanking the central tetra-nucleotide motif in State 1’ is poor, precluding reliable model building (Method and Extended Data Fig. 7a). We therefore only built models for State 1 and State 2, which also represent the most extreme discrete dimer configurations (Method and Extended Data Fig. 7). The overall structure of the A3G-VCBC monomer is the same in the different dimeric configurations, as the maximal pairwise backbone RMSD is ~ 0.5 Å between monomer subunits. The 5' end of the single-stranded RNA binds an A3G-VCBC interface in the monomer whereas the 3' end extends into the next copy bridging the monomers to form dimers (Extended Data Fig. 7a). The four-nucleotide core (NT1-4) is conformationally preserved between monomer copies of A3G-RNA-VCBC. In addition, the nucleotides flanking this core sequence are in different conformations in State 1 and State 2 and may reflect structural plasticity of RNA, which gives rise to the different E3 ligase receptor conformational states (Extended Data Fig. 8a-c).

Additional protein surfaces are buried by dimerization of A3G-VCBC subunits in State 1 and State 2 (Extended Data Fig. 8d,e). In State 1, self-association of A3G is mediated by cation-pi interactions between R29 and Y13 and salt bridges between R30 and D15 (Extended Data Fig. 8d). Interestingly, the R30A mutation of human A3G reduces self-association, encapsidation and restriction of HIV-1[11,97]. Therefore, the interaction mode of Vif, RNA and A3G in State 1 is consistent with our conclusion that Vif binds A3G/RNA in a manner that limits A3G escape over

long evolutionary timescales by engaging an essential surface required for antiviral function (see "Evolution of the Vif-A3G interface", Main Text). Compared to State 1, State 2 has almost no A3G-A3G contacts; instead, A3G makes a few interactions with Vif from the second copy of A3G-VCBC (Extended Data Fig. 8e), consistent with notion that Vif disrupts A3G self-association and packaging independent of ubiquitin transfer. Both State 1 and State 2 are compatible with ubiquitination of A3G, as the dimeric configuration of A3G-VCBC are related by a simple rigid body rotation and translation, which does not affect how the ubiquitin acceptor lysines on CDA2 of A3G are presented to coenzymes such as ARIH2 bound to CRL5 (Extended Data Fig. 8f and Supplementary Video 1).

We speculate that the discrete dimerization states of A3G-RNA-VCBC represent two of many configurations that could assemble on RNAs of different nucleotide sequences flanking the conformationally conserved four nucleotide core (NT1-4) (Extended Data Fig. 7a). However, we cannot rule out a model where the discrete states of A3G-VCBC possess distinct functional roles. The significance of dimeric assemblies of A3G-VCBC for viral infectivity requires further investigation.

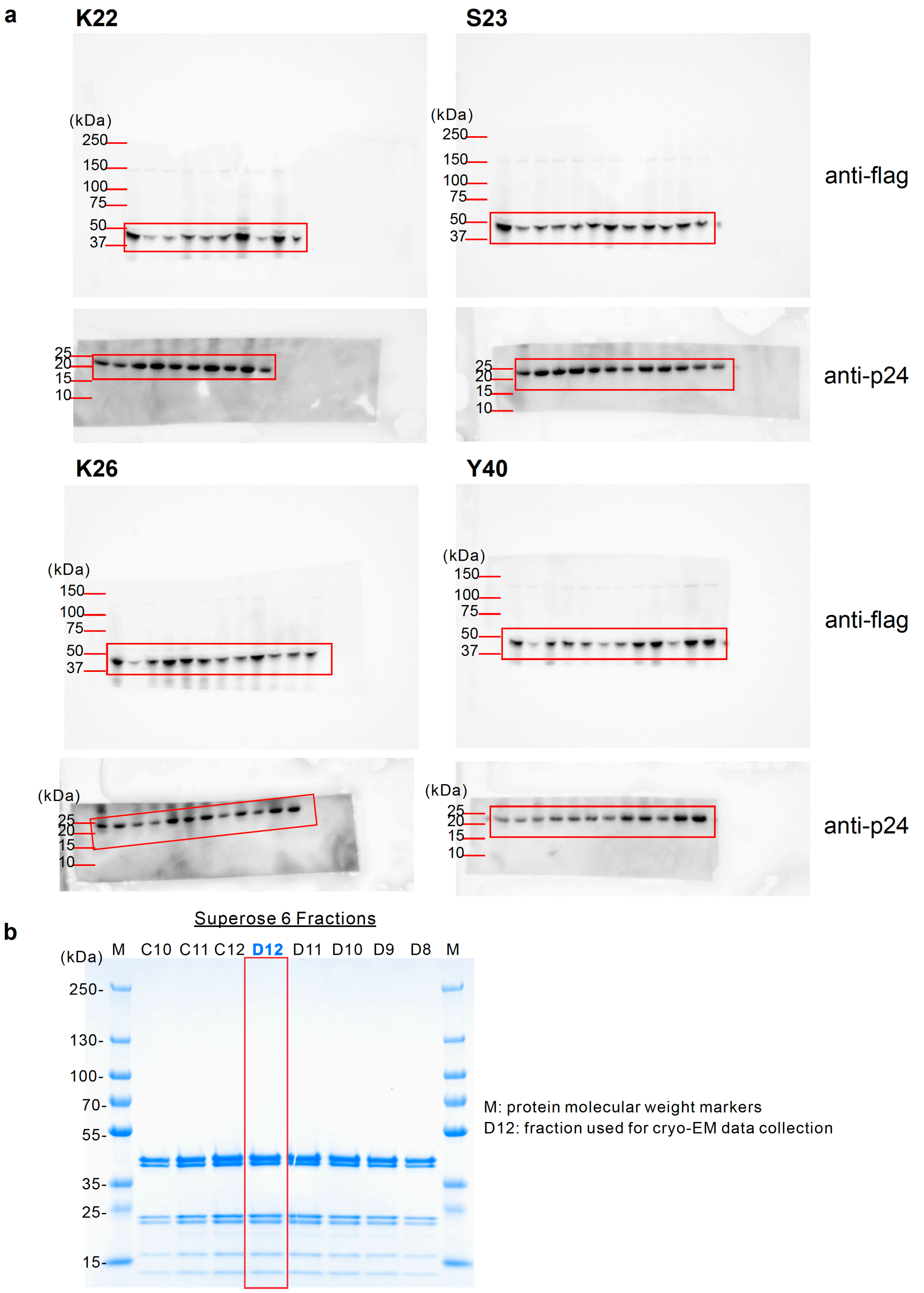

Supplementary Figure 1 | Uncropped source images. a, Source western blots related to Fig. 1f and Extended Data Fig. 5. Controls were run on the same gel as the samples. **b,** Source coomassie blue-stained SDS-PAGE related to Extended Data Fig. 1a. Red square indicates how the images were cropped for the final figure.

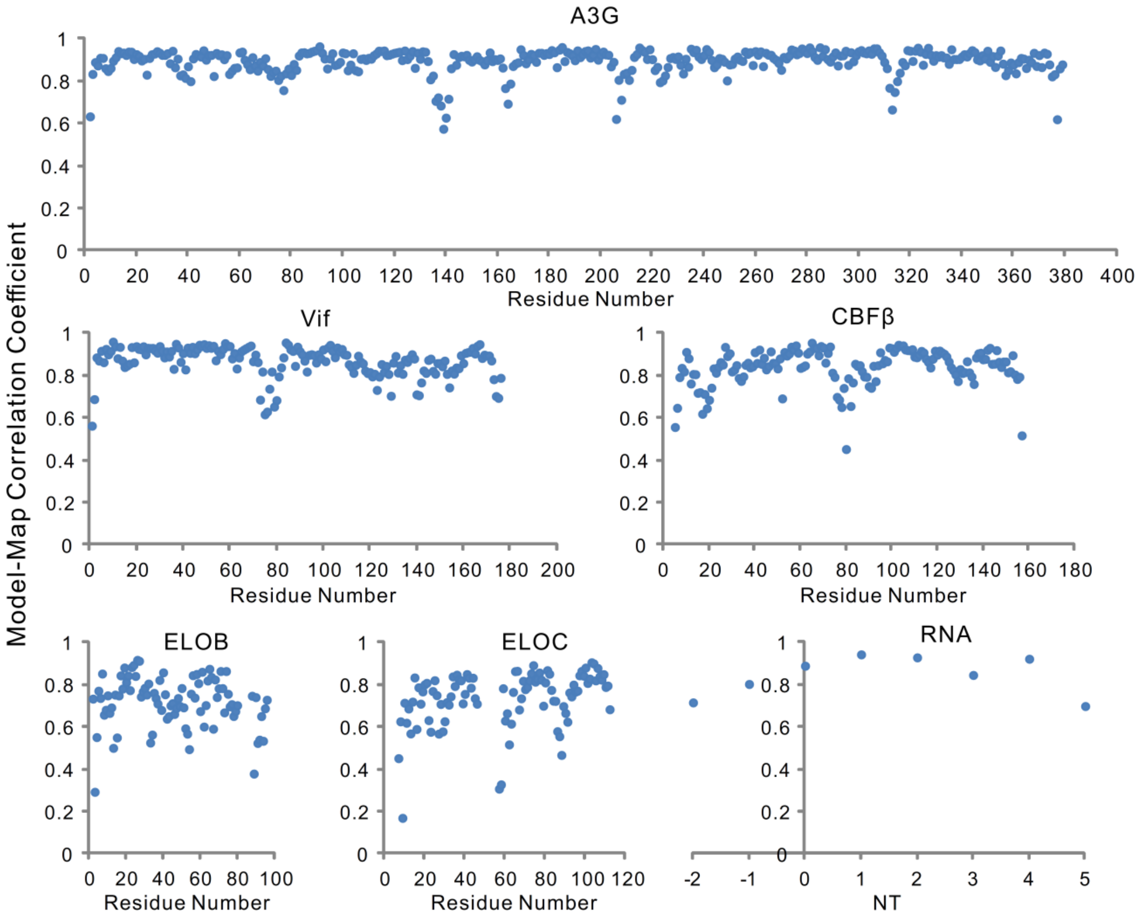

Supplementary Figure 2 | Model-map fit analysis of A3G-RNA-VCBC monomeric complex. Related to Extended Data Fig. 3. The y-axis is correlation coefficient calculated by PHENIX[86]. The x-axis is residue number and nucleotide (NT) number for protein and RNA, respectively

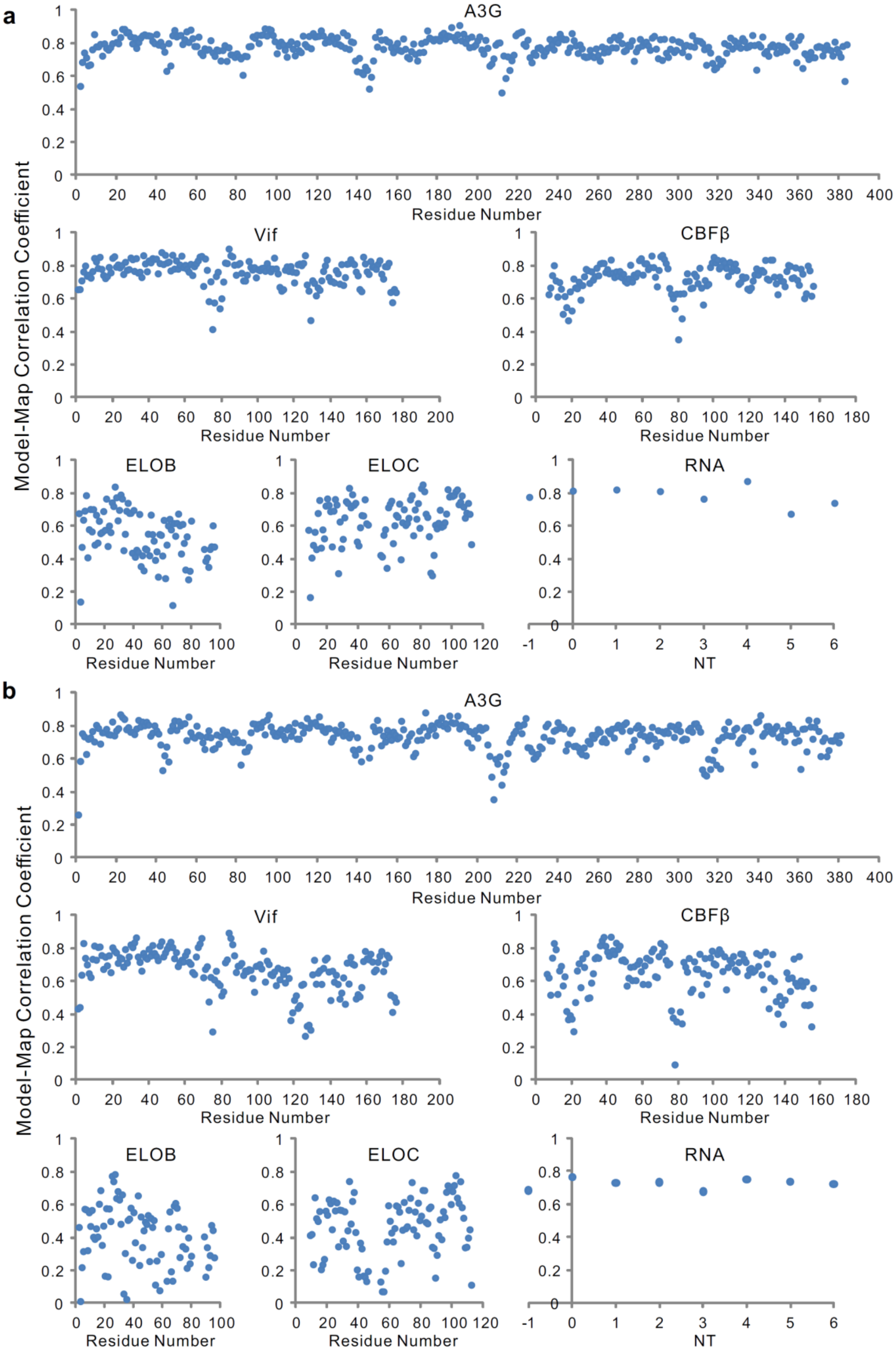

Supplementary Figure 3 | Model-map fit analysis of A3G-RNA-VCBC dimeric complex in State 1. Related to Extended Data Fig. 3. Panel **a** and **b** represent different A3G-RNA-VCBC copies. The y-axis is correlation coefficient calculated by PHENIX[86]. The x-axis is residue number and nucleotide (NT) number for protein and RNA, respectively.

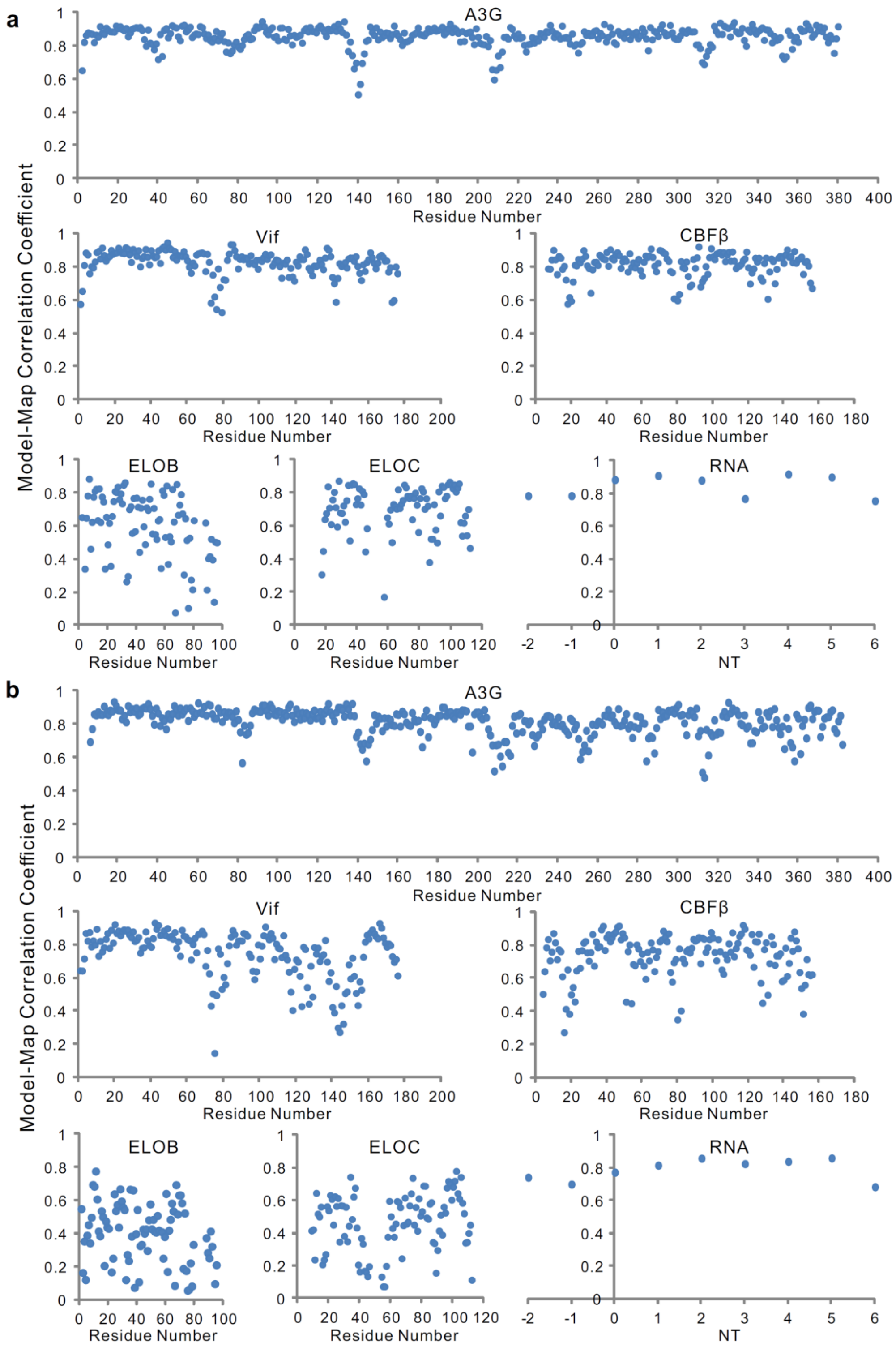

Supplementary Figure 4 | Model-map fit analysis of A3G-RNA-VCBC dimeric complex in State 2. Related to Extended Data Fig. 3. Panel **a** and **b** represent different A3G-RNA-VCBC copies. The y-axis is correlation coefficient calculated by PHENIX[86]. The x-axis is residue number and nucleotide (NT) number for protein and RNA, respectively.

Supplementary Table 1 | Summary of Vif residues interacting with RNA

	**Identified in A3G-RNA-VCBC structure in this study**				**Functionally verified in literature**		
Residue	Monomer	Dimer State 1	Dimer State 2	NT	RNA binding[b]	A3G antagonism[a,b]	Reference
R19					ND	**Yes**	[11]
T20					ND	ND	
K22					ND	**Yes**	[35,42,98,99]
S23					ND	No (S23A, NL43)<br>No (R23A, LAI)	[42,49]
K26					**Yes**	**Yes**	[42,49,98,100]
H27					ND	No (H27Y; LAI)	[49]
Y30					**Yes**	**Yes**	[42,100]
Y40					**Yes**	**Yes**	[35,40,49,99,101]
H42					ND	**Yes**	[35,42,49]
H43					**Yes**	**Yes**	[35,49,100]
Y44					**Yes**	**Yes**	[35,49,100]

[a] ND: not determined

[b] No (mutated residue, virus strain): No effect on A3G antagonism with indicated mutation on HIV-1 Vif

Interaction observed in monomer and both copies of dimers in State 1 and State 2

Interaction only observed in one monomer of State 1 or State 2

Interaction not detected

Interaction with core tetra-nucleotides NT1-4

Interaction with terminal nucleotides other than NT1-4

Supplementary Table 2 | Summary of A3G residues interacting with RNA

	**Identified in A3G-RNA-VCBC structure in this study**				**Functionally verified in literature[c,d]**			
Residue[a,b]	Monomer	Dimer State 1	Dimer State 2	NT	RNA/A3G Association	A3G Packaging	HIV-1 Restriction	Reference
Y13					ND	ND	ND	
R24					**Yes**	**Yes**	**Yes**	11,97
P25	C=O	C=O	C=O		ND	ND	ND	
I26					**Yes**	**Yes**	ND	97
L27					**Yes**	ND	ND	102
S28					**Yes**	**Yes**	**Yes**	34,97
R29					**Yes**[e]	No (R29A)	ND	97,103
N31					No (N31S)	ND	ND	103
V58					ND	ND	ND	
Y59					**Yes**	ND	ND	102
W94					**Yes**	**Yes**	**Yes**	11,34,97,104
L123	C=O	C=O	C=O		**Yes**	No (L123A)	No (L123A)	12,105
Y124					**Yes**	**Yes**	**Yes**	12,34,97,104,105
Y125	N-H	N-H	N-H		**Yes**	**Yes**	**Yes**	12,34,49,105
F126					**Yes**	**Yes**	**Yes**	12,34,97,105
W127					**Yes**	**Yes**	**Yes**	11,12,34,97,104,105
F268[f]	C=O	C=O	C=O		ND	ND	ND	
K270[f]					**Yes (partial)**	ND	ND	102

[a] Residues interacting with 5'end of RNA (NT-1 and NT-2, Extended Data Fig. 9a) are not listed due to relatively poor local resolution (Extended Data Figs. 4c,9c).
[b] A3G residues (V9, R11, T16, W34, R55) identified to interact with NT6 in State 1 and State 2 are not included in the table because the local density of NT6 is not as well-resolved as nearby nucleotides.
[c] ND: not determined
[d] No (mutated residue): No effect with indicated mutation on hA3G
[e] Double mutation R29S/R30S
[f] A double mutation F268N/K270E on A3G abrogated A3G-Vif interaction in co-IP experiment[11]

Interaction observed in monomer and both copies of dimer in State 1 and State 2
Interaction only observed in one monomer of State 1 and State 2
Interaction not detected
Residues from another A3G copy in the dimer
C=O Backbone carbonyl (instead of side chain) participates in RNA interaction
N-H Both backbone amide and side chain participate in RNA interaction
Interaction with core tetra-nucleotide NT1-4
Interaction with terminal nucleotides other than NT1-4

SUPPLEMENTARY INFORMATION REFERENCES

97 Fukuda, H. *et al.* Structural Determinants of the APOBEC3G N-Terminal Domain for HIV-1 RNA Association. *Front Cell Infect Microbiol* **9**, 129, doi:10.3389/fcimb.2019.00129 (2019).

98 Dang, Y., Wang, X., Zhou, T., York, I. A. & Zheng, Y. H. Identification of a novel WxSLVK motif in the N terminus of human immunodeficiency virus and simian immunodeficiency virus Vif that is critical for APOBEC3G and APOBEC3F neutralization. *J Virol* **83**, 8544-8552, doi:10.1128/JVI.00651-09 (2009).

99 Simon, V. *et al.* Natural variation in Vif: differential impact on APOBEC3G/3F and a potential role in HIV-1 diversification. *PLoS Pathog* **1**, e6, doi:10.1371/journal.ppat.0010006 (2005).

100 Zhang, H., Pomerantz, R. J., Dornadula, G. & Sun, Y. Human immunodeficiency virus type 1 Vif protein is an integral component of an mRNP complex of viral RNA and could be involved in the viral RNA folding and packaging process. *J Virol* **74**, 8252-8261, doi:10.1128/jvi.74.18.8252-8261.2000 (2000).

101 Fang, L. & Landau, N. R. Analysis of Vif-induced APOBEC3G degradation using an alpha-complementation assay. *Virology* **359**, 162-169, doi:10.1016/j.virol.2006.09.013 (2007).

102 Uyttendaele, I. *et al.* Random mutagenesis MAPPIT analysis identifies binding sites for Vif and Gag in both cytidine deaminase domains of Apobec3G. *PLoS One* **7**, e44143, doi:10.1371/journal.pone.0044143 (2012).

103 Lavens, D. *et al.* Definition of the interacting interfaces of Apobec3G and HIV-1 Vif using MAPPIT mutagenesis analysis. *Nucleic Acids Res* **38**, 1902-1912, doi:10.1093/nar/gkp1154 (2010).

104 Bach, D. *et al.* Characterization of APOBEC3G binding to 7SL RNA. *Retrovirology* **5**, 54, doi:10.1186/1742-4690-5-54 (2008).

105 Zhang, W. *et al.* Association of potent human antiviral cytidine deaminases with 7SL RNA and viral RNP in HIV-1 virions. *J Virol* **84**, 12903-12913, doi:10.1128/JVI.01632-10 (2010).
