# Supplementary material for: The structural basis for HIV-1 Vif antagonism of human APOBEC3G
Source: Nature. 2023 Feb 8;615(7953):728–33. doi: 10.1038/s41586-023-05779-1 (PMC10033410; doi:10.1038/s41586-023-05779-1)
Supplement: Supplementary file 2 — Reporting Summary [file 41586_2023_5779_MOESM2_ESM.pdf]

nature portfolio

Corresponding author(s): John D. Gross

Last updated by author(s): 2022 - 12 - 14

Reporting Summary

Nature Portfolio wishes to improve the reproducibility of the work that we publish. This form provides structure for consistency and transparency in reporting. For further information on Nature Portfolio policies, see our Editorial Policies and the Editorial Policy Checklist.

Statistics

For all statistical analyses, confirm that the following items are present in the figure legend, table legend, main text, or Methods section.

n/a	Confirmed	
☐	☒	The exact sample size (*n*) for each experimental group/condition, given as a discrete number and unit of measurement
☐	☒	A statement on whether measurements were taken from distinct samples or whether the same sample was measured repeatedly
☒	☐	The statistical test(s) used AND whether they are one- or two-sided<br>*Only common tests should be described solely by name; describe more complex techniques in the Methods section.*
☒	☐	A description of all covariates tested
☒	☐	A description of any assumptions or corrections, such as tests of normality and adjustment for multiple comparisons
☐	☒	A full description of the statistical parameters including central tendency (e.g. means) or other basic estimates (e.g. regression coefficient) AND variation (e.g. standard deviation) or associated estimates of uncertainty (e.g. confidence intervals)
☒	☐	For null hypothesis testing, the test statistic (e.g. *F*, *t*, *r*) with confidence intervals, effect sizes, degrees of freedom and *P* value noted<br>*Give P values as exact values whenever suitable.*
☒	☐	For Bayesian analysis, information on the choice of priors and Markov chain Monte Carlo settings
☒	☐	For hierarchical and complex designs, identification of the appropriate level for tests and full reporting of outcomes
☒	☐	Estimates of effect sizes (e.g. Cohen's *d*, Pearson's *r*), indicating how they were calculated

Our web collection on statistics for biologists contains articles on many of the points above.

Software and code

Policy information about availability of computer code

Data collection	SerialEM (v.3.8.6)
Data analysis	MotionCor2 (v.1.4.3), CryoSPARC (v.3.0-3.2), RELION (v.3.0-3.1), PHENIX (v1.14; v.1.19.2), Coot (v.0.9.1), ISOLDE (v.1.0.1), Chimera (v.1.14-1.16), Chimera X (v.1.4), ResMap (v.1.95), PyEM (v.0.5), MODELLER (v10.1), BINANA (v2.0), PLIP server, MolProbity (v.4.02), WebLogo, ESPript (v3), PyMOL (v.2.5.2), AlphaFold 2

For manuscripts utilizing custom algorithms or software that are central to the research but not yet described in published literature, software must be made available to editors and reviewers. We strongly encourage code deposition in a community repository (e.g. GitHub). See the Nature Portfolio guidelines for submitting code & software for further information.

Data

Policy information about availability of data

All manuscripts must include a data availability statement. This statement should provide the following information, where applicable:

- Accession codes, unique identifiers, or web links for publicly available datasets
- A description of any restrictions on data availability
- For clinical datasets or third party data, please ensure that the statement adheres to our policy

EM maps for A3G-VCBC monomeric complex are deposited in the Electron Microscopy Data Bank (EMDB) under the accession number EMD-27032, and the associated atomic coordinate is deposited in the Protein Data Bank (PDB) under the accession code 8CX0. EM maps for A3G-VCBC dimeric complex in State 1 are deposited in the EMDB under the accession number EMD-27033, and the associated atomic coordinate is deposited in the PDB under the accession code 8CX1. EM maps for A3G-VCBC dimeric complex in State 2 are deposited in the EMDB under the accession number EMD-27034, and the associated atomic coordinate is deposited in the PDB under the accession code 8CX2. EM maps for A3G-VCBC dimeric complex in State 1' are deposited in the EMDB under the accession number

EMD-28667. Coordinate files from previous publication used for the initial model building into the cryo-EM maps and comparative model building are AlphaFold 2 Q9HC16 (hA3G), PDB 4N9F (VCBC-CUL5NTD), PDB 6P59 (SIVrcm VCBC), PDB 7ONI (NEDD8-CUL5CTD-RBX2-ARIH2), and partial structure CUL1-RBX1-Ub-ARIH1 from PDB 7B5M.

Field-specific reporting

Please select the one below that is the best fit for your research. If you are not sure, read the appropriate sections before making your selection.

☒ Life sciences ☐ Behavioural & social sciences ☐ Ecological, evolutionary & environmental sciences

For a reference copy of the document with all sections, see nature.com/documents/nr-reporting-summary-flat.pdf

Life sciences study design

All studies must disclose on these points even when the disclosure is negative.

Sample size	The appropriate sample size was not predetermined in cryo-EM data analysis. The number of micrographs were increased until the satisfactory map quality and resolution was achieved. The number of micrographs collected was 6429 and the number of particles that went into the final reconstruction were 495,571, 57,207, 46,083, and 51,055 for monomer, dimer in State 1, State 2 and State 1', which resulted in 2.5-3.5 Å overall resolution (FSC=0.143) and was sufficient to provide well-resolved structural detail.
Data exclusions	Micrographs with resolution poorer than 4 Å and excessive ice contamination were excluded. Particle images that could not be classified into useful reconstructions were discarded during cryo-EM data processing as it was a required procedure to maximize the final map quality and resolution as described in this study.
Replication	A3G degradation assay was performed with two biological replicates, independent assays. A3G-VCBC-CUL5N purification was repeated for at least 3 times and all attempts at replication were successful. Negative stain EM and cryo-EM images collected on different days and with different protein preparations yielded congruent results. Duplicate grids were frozen from the same preparation on both UltraAuFoil and Quantifoil grids and used for initial screening. 6429 micrographs collected from UltraAuFoil grid on the Titan Krios electron microscope was used for our final dataset. Quantifoil grids yielded suboptimal ice thickness, but comparable particle distribution. Quantifoil grids reproduced consistent 2D class averages as UltraAuFoil grids but showed preferred orientation and thus not included in this study.
Randomization	All cryo-EM particle images were randomly split into two half dataset for the estimation of overall resolution during Fourier Shell Correlation calculation. Otherwise, randomization was not relevant to this study.
Blinding	Blinding was not relevent to this study

Reporting for specific materials, systems and methods

We require information from authors about some types of materials, experimental systems and methods used in many studies. Here, indicate whether each material, system or method listed is relevant to your study. If you are not sure if a list item applies to your research, read the appropriate section before selecting a response.

Materials & experimental systems

n/a	Involved in the study	
☐	☒	Antibodies
☐	☒	Eukaryotic cell lines
☒	☐	Palaeontology and archaeology
☒	☐	Animals and other organisms
☒	☐	Human research participants
☒	☐	Clinical data
☒	☐	Dual use research of concern

Methods

n/a	Involved in the study	
☒	☐	ChIP-seq
☒	☐	Flow cytometry
☒	☐	MRI-based neuroimaging

Antibodies

Antibodies used	Monoclonal ANTI-FLAG® M2 antibody produced in mouse, Sigma-Aldrich F1804. ARP-3537 Anti-Human Immunodeficiency Virus 1 (HIV-1) p24 Monoclonal (183-H12-5C). Home / IgG / Mouse IgG HRP-conjugated Antibody HAF007, bio-techne.
Validation	Not applicable

Eukaryotic cell lines

Policy information about cell lines

Cell line source(s)	Sf9 insect cells (Thermo Fisher Scientific; Gibco cat.12659017), HEK293T cells (ATCC CRL-3216)

Authentication	Cell lines were not authenticated
Mycoplasma contamination	All cells were tested negative for mycoplasma contamination by the authors of this study.
Commonly misidentified lines (See ICLAC register)	No commonly misidentified cells lines were used in this study
